# *Schistosoma mansoni* eggs induce Wnt/β-catenin signaling and activate the protooncogene c-Jun in human and hamster colon

**DOI:** 10.1038/s41598-020-79450-4

**Published:** 2020-12-23

**Authors:** Jakob Weglage, Friederike Wolters, Laura Hehr, Jakob Lichtenberger, Celina Wulz, Felix Hempel, Anne Baier, Thomas Quack, Kernt Köhler, Thomas Longerich, Gabriele Schramm, Karuna Irungbam, Heike Mueller, Verena von Buelow, Annette Tschuschner, Margarete Odenthal, Uta Drebber, Maha El Arousy, Leandra N. Z. Ramalho, Katrin Bankov, Peter Wild, Jörn Pons-Kühnemann, Jonas Tschammer, Christoph G. Grevelding, Elke Roeb, Martin Roderfeld

**Affiliations:** 1 Department of Gastroenterology, Justus Liebig University, Gaffkystr. 11, 35392 Giessen, Germany; 2 Institute of Parasitology, BFS, Justus Liebig University, Giessen, Germany; 3 Institute of Veterinary Pathology, Justus Liebig University, Giessen, Germany; 4 Translational Gastrointestinal Pathology, Institute of Pathology, University Hospital Heidelberg, Heidelberg, Germany; 5 Experimental Pneumology, Priority Research Area Asthma and Allergy, Research Center Borstel, Borstel, Germany; 6 Institute of Pathology, University of Cologne, Cologne, Germany; 7 Center of Molecular Medicine (CMMC), University of Cologne, Cologne, Germany; 8 Department of Parasitology, Faculty of Medicine, Cairo University, Cairo, Egypt; 9 Department of Pathology, Ribeirão Preto Medical School, University of São Paulo, São Paulo, Brazil; 10 Dr. Senckenberg Institute of Pathology, University Hospital Frankfurt, Frankfurt am Main, Germany; 11 Institute of Medical Informatics, Justus Liebig University, Giessen, Germany

**Keywords:** Infection, Colonic diseases

## Abstract

Schistosomiasis (bilharzia) is a neglected tropical disease caused by parasitic flatworms of the genus *Schistosoma*, with considerable morbidity in parts of the Middle East, South America, Southeast Asia, in sub-Saharan Africa, and particularly also in Europe. The WHO describes an increasing global health burden with more than 290 million people threatened by the disease and a potential to spread into regions with temperate climates like Corsica, France. The aim of our study was to investigate the influence of *S. mansoni* infection on colorectal carcinogenic signaling pathways in vivo and in vitro. *S. mansoni* infection, soluble egg antigens (SEA) and the Interleukin-4-inducing principle from *S. mansoni* eggs induce Wnt/β-catenin signaling and the protooncogene c-Jun as well as downstream factor Cyclin D1 and markers for DNA-damage, such as Parp1 and γH2a.x in enterocytes. The presence of these characteristic hallmarks of colorectal carcinogenesis was confirmed in colon biopsies from *S. mansoni*-infected patients demonstrating the clinical relevance of our findings. For the first time it was shown that *S. mansoni* SEA may be involved in the induction of colorectal carcinoma-associated signaling pathways.

## Introduction

Schistosomiasis is one of the most prevalent parasitic diseases worldwide with at least 290 million people requiring preventive treatment in 2018^1^. While the majority of people at risk live in the endemic regions of Africa, *Schistosoma* species are also prevalent in the Middle East, the Caribbean, South America, and South East Asia^2^. Schistosomiasis is increasingly imported into regions with temperate climates by immigrants and travelers from endemic areas^3,4^. A recent epidemiological case study, investigating an outbreak of urogenital schistosomiasis in Corsica, France, emphasized the potential risk of schistosomiasis spreading into novel areas^5^. It has been suggested that the persistent establishment of the tropical disease in Europe relies on the preadaptation of *schistosomes* and the adaptation of secondary hosts to overwinter in countries like France, Italy, Portugal, Spain, and Greece^6^.

In humans, intestinal schistosomiasis is caused by different species such as *Schistosoma mansoni (S. mansoni)* and *S. japonicum*^7^. During infection, the paired adult worms lodge in the mesenteric veins and produce approximately 300 eggs (ova) per day^8^. About half of the eggs migrate through the intestinal wall into the gut lumen and are excreted with the feces, thus completing the parasites life cycle^9^. Without providing any obvious intrinsic motility mechanisms, schistosome eggs are likely to depend on host-driven processes to pass tissues^10^. In the colorectal wall, ova induce an inflammatory response, which can lead to fibrotic granuloma formation and lymphoid hyperplasia, resulting in ova entrapment^7^. Eggs deposited in living host tissues might survive for up to three months^11^. Being damaged by the eggs’ extravasation, the overlying mucosa develops small superficial ulcers with hyperplastic changes^7^. During chronification, this process provokes mucosa hypertrophy and subsequent pseudopolyp formation^12^. However, about half of the eggs, instead of reaching the intestinal lumen, are swept into the liver, provoking marked granulomatous inflammation^10^. Typical symptoms are abdominal cramping, diarrhea, and dysentery^13^. Schistosomiasis is diagnosed by the detection of parasite eggs in stool (*S. mansoni*, *S. japonicum*) or urine (*S. haematobium*) samples or, if accessible, by serological and immunological tests^1^.

The global burden of cancer was estimated to be 18.1 million new cases and 9.6 million cancer-related deaths in 2018^14^. Approximately 20% of human cancers are caused by infectious diseases^15^. It was estimated, that 0.4% of the new cancers that are attributable to infections were caused by the trematodes *Schistosoma haematobium* (0.3%), *Opisthorchis viverrini*, and *Clonorchis sinensis* (both liver flukes together: 0.1%), which are considered as group 1 carcinogens by IARC^16^. The infection with the liver flukes *O. viverrini* and *C.* increase the risk to develop cholangiocarcinoma, while in endemic areas, 46–75% of all bladder cancers were attributed to *S. haematobium*^17^. Clinical studies and animal experiments have also indicated that schistosomiasis promotes the development of hepatocellular carcinoma (HCC), prostate cancer, follicular lymphomas, and also colorectal cancer (CRC)^18–21^. A recent study has suggested that schistosomal colitis is more commonly associated with an earlier onset of multicentric CRC, high percentage of mucinous adenocarcinoma, and presents at an advanced stage^19^. Another recently published report demonstrated that *S. japonicum* infection facilitates the development of CRC, particularly in the early and intermediate stages, suggesting that schistosomiasis may alter the mechanisms underlying the progression of CRC^22^. Nevertheless patients with schistosomal and nonschistosomal CRC, had the same outcome after 7.5 years^22^. Although numerous studies have demonstrated an association of *S. mansoni* infection with liver cancer, colorectal carcinoma, and bladder cancer^17^, no reliable quantitative data to substantiate this relationship are available, yet.

Lastly, in contrast to schistosomiasis-related carcinogenesis, the link between inflammatory bowel disease (IBD) and CRC has been investigated extensively. A recent meta-analysis, including 7199 cases of IBD, demonstrated that patients with long lasting ulcerative colitis (UC) and colon-associated Crohn's disease (CD) have an increased risk of CRC^23^. While CD is associated with T-helper type 1 (Th1) immune responses, UC shows a Th2-prone fibroinflammatory phenotype^24^. *S. mansoni* egg-derived antigens also induce colitis characterized by a Th2-prone fibroinflammatory phenotype which may contribute for increasing the susceptibility to oncogenic provocations^2,10^.

Previous research has, moreover, provided first insights into the molecular mechanisms underlying *Schistosoma*-associated carcinogenesis. It has been postulated that genotoxic agents, produced by the eggs, may play a role in CRC via alteration of the Bcl-2 protein expression pattern, leading to a dysregulation of apoptosis^25^. The glycoprotein *IL-4-inducing principle from Schistosoma mansoni eggs* (IPSE) is the major egg secreted product with immunoglobulin-binding properties^26^. The interaction of IgE bound IPSE with the FcεRI receptor can trigger the release of IL-4 and IL-13 from basophils and is therefore involved in immunemodulation during schistosomiasis^27^. Moreover, IPSE/alpha-1 has a C-terminal functional nuclear localization sequence (NLS) for translocation into the nucleus, which suggests a possible function as transcription factor^28^.

Similar to what has been described in various cancer entities, the growth of new blood vessels was enhanced in *S. mansoni* infection^10^ and it was suggested that the eggs might induce angiogenesis-related processes by up-regulating vascular endothelial growth factor and creating a proangiogenic environment, characterized by hypoxia, acidic pH, and low glucose concentration, due to vessel occlusion^29^.

Based on these findings, we hypothesize that *S. mansoni*-associated colitis could promote the development of CRC. Therefore, we analyzed CRC-associated signaling pathways and CRC markers in colon samples from *S. mansoni*-infected patients, from *S. mansoni*-infected hamsters, and from SEA-stimulated enterocytes.

## Materials and methods

### Human material

Pseudonymized human colon samples were kindly provided by cooperation partners (TL, MO, UD, MA, LR, KB, and PW) after approval by the local ethics committee (Ethics vote ID AZ 05/19, Justus Liebig University Giessen). According to the ethics vote, an informed consent was not required for our retrospective analyses of archived tissues. It was not appropriate or possible to involve patients or the public in the design, or conduct, or reporting, or dissemination plans of our research.

### Animal model

According to the principles of the 3Rs, colon samples of female hamsters that were destined for maintaining the *S. mansoni* life cycle, were utilized for this study. To keep the *S. mansoni* life cycle, *Biomphalaria glabrata* snails served as intermediate hosts and Syrian hamsters (*Mesocricetus auratus*) as final hosts. The strain of *S. mansoni* originated from a Liberian isolate obtained from Bayer AG (Monheim, Germany). Bisex (= mixed sex) and single-sex (= unisexual) worm populations were generated by polymiracidial and monomiracidial intermediate host infections, respectively^30^. All animal experiments were performed in accordance with the European Convention for the Protection of Vertebrate Animals used for experimental and other scientific purposes (ETS No 123; revised Appendix A) and have been approved by the Regional Council Giessen (approval number V54-19 c 20/15 c GI 18/10 Nr. A1/2014).

### Isolation of soluble egg antigens

*Schistosoma mansoni* eggs were obtained from livers of bisex-infected hamsters at day 46 post infection and soluble egg antigens (SEA) were isolated as described earlier^27^. SEA protein concentration was determined colorimetrically, utilizing the Advanced Protein Assay (Cytoskeleton, Inc., Denver, USA) according to the manufacturer's instructions.

### Purification of nIPSE/alpha-1 and HEK-IPSE

Natural (n)IPSE/alpha-1 (Interleukin-4 inducing principle from *S. mansoni* eggs) was purified from SEA in a two-step chromatography procedure including a cation exchange chromatography followed by an affinity chromatography via binding to monoclonal anti-IPSE/alpha-1 antibodies coupled to NHS-activated Sepharose as described before^27^. The eluted neutralized fractions were controlled by SDS-PAGE, silver staining, and western blot for content and purity of IPSE/alpha-1, dialyzed against PBS (pH 7.5), concentrated to a suitable concentration, and stored in aliquots at − 80 °C. Human embryonic kidney (HEK)-IPSE was purified from 293 HEK cells transfected with the expression vector pSecTag2-IPSE. Secreted HEK-IPSE (His-tagged) was sequentially purified from the culture medium (10% FCS / RPMI, Capricorn) by immobilized metal affinity chromatography and antibody affinity chromatography. Eluted fractions were treated as described above for the purification of nIPSE.

### Cell culture experiments

SW620 cells (ATCC Cat# CCL-227, RRID:CVCL_0547) were stimulated in vitro at 80% confluency with 1–15 µg/ml SEA or IPSE with denoted concentrations and for the indicated time. SEA contains around 1–2% IPSE (personal communication by G. Schramm). The concentrations of SEA and IPSE were titrated to demonstrate similar levels of activation in blots showing both, SEA and IPSE. Inhibition experiments were conducted after starving the cells overnight in cell culture medium without FCS. The cells were pretreated with SP600125 or U0126 (both 10 µM, 30 min) or XAV939 (11 nM, overnight) before stimulation.

### Phospho-kinase proteome profiler array

Lysates were prepared according to the manufacturer’s protocol from SEA- and mock-stimulated SW620 cells. The human Phospho-Kinase Proteome Profiler Array (R&D Systems, Minneapolis, MN, USA) were carried out as indicated in the manual. Densitometric analysis was performed as described previously^31^.

### Immunohistochemistry

Detections of c-Jun (Cell Signaling Technology, Cat# 9165, RRID:AB_2130165), β-catenin (Cell Signaling Technology Cat# 9581, RRID:AB_490891), Mcm2 (Cell Signaling Technology, Cat# 4007, RRID:AB_2142134), γ-H2a.x (Cell Signaling Technology, Cat# 9718, RRID:AB_2118009), Ki67 (Agilent Cat# M7240, RRID:AB_2142367), and Cyclin D1 (Santa Cruz Biotechnology, Heidelberg, Germany, Cat# sc-20044, RRID:AB_627346) were performed as described^32, 33^. Appropriately concentrated antibodies were used for isotype controls.

### Western blot analysis

Protein samples were prepared from total colon lysates or cultured cells, boiled in Laemmli-buffer for 5 min, chilled on ice, subjected to 10% SDS-PAGE and transferred to polyvinylidene difluoride membranes. The aforementioned- and the following antibodies were used for specific detection of the analytes: phospho-c-Jun (Cell Signaling Technology Cat# 3270, RRID:AB_2129575), phospho-Gsk3β^Ser9^ (Cell Signaling Technology Cat# 9323, RRID:AB_2115201), phospho-β-catenin (Cell Signaling Technology Cat# 9561, RRID:AB_331729), Parp1 (Cell Signaling Technology Cat# 9542, RRID:AB_2160739), α-Tubulin (Cell Signaling Technology Cat# 2144, RRID:AB_2210548), Gapdh (Proteintech Cat# 60004-1-Ig, RRID:AB_2107436). Visualization of proteins was performed by Horseradish-Peroxidase (HRP)-linked secondary antibodies and the ECL Chemiluminescence Detection Kit (SuperSignal West Pico Chemiluminescent Substrate, Thermo Fisher Scientific, Waltham, MA, USA) according to the manufacturer’s protocols. Semiquantitative analysis of obtained signals was performed utilizing ImageJ Software, RRID: SCR_003070.

### Reporter gene assay

For analysis of the AP-1 transcriptional promotor activity, SW620 cells were transfected with pGL4.44[luc2P/AP1 RE/Hygro] Vector (Promega, Walldorf, Germany, Cat# E4111). The pGL4.74[hRluc/TK] plasmid vector (Promega, Cat# E692A) has been used as a control of transfection efficiency. Cells were treated with SEA, nIPSE, HEK-IPSE or PBS as indicated. The Dual Glo Luciferase assay system (Promega, Cat#, E2940) has been performed according to the manufacture’s protocol. Chemiluminescence of *Firefly* and *Renilla* luciferase activity has been measured with a Tecan plate reader (Tecan, Männedorf, Switzerland, Infinite M Plex).

### Comet assay

The alkaline comet assay (Abcam Cat# ab238544) has been performed for assessing DNA damage in SW620 cells. SW620 cells were stimulated for 48 h with PBS or SEA as indicated. The assay was performed according to the manufacturer’s protocol.

### Statistical analysis

Statistical analysis was performed using SPSS version 26.0 (SPSS, IBM, Armonk, NY, RRID:SCR_002865). Mann–Whitney U test^34^ was applied for hypothesis testing to evaluate expression and phosphorylation levels in western blot analyses of hamster samples. The significance level ⍺ was set to 0.05. *P* Values were adjusted by the Bonferroni–Holm method^35^. Significant differences are labeled by an asterisk. Densitometrically assessed data from western blots (hamster colon) are depicted as means or median ± 95% confidence intervals.

## Results

### *Schistosoma mansoni* eggs induced Wnt/β-catenin-signaling in colon epithelial cells

**Figure 1:**
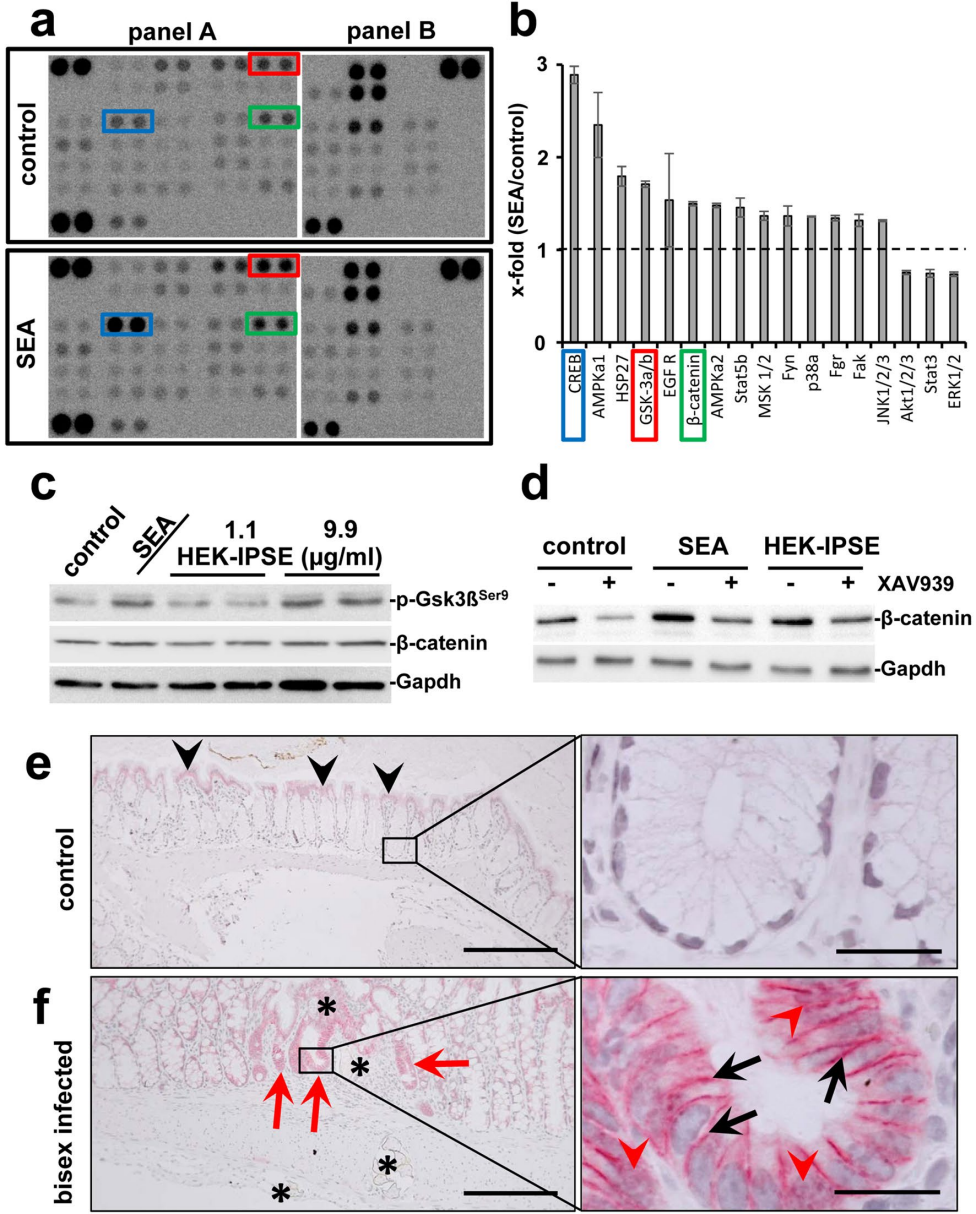
Eggs of S. mansoni induce Wnt-signaling/ß-Catenin in colon epithelium. (**a**) Phosphokinase proteome profiler arrays and (**b**) densitometric assessment thereof demonstrated the induction of β-catenin as well as the phosphorylation of Gsk3β and CREB in SW620 cells after soluble egg antigen (SEA)-stimulation. The 17 most regulated factors of the array are depicted in (**b**). The Human Phospho-Kinase Array is a tool to simultaneously detect the relative levels of phosphorylation of kinases and two related total proteins. The array consists of two membranes (left and right membrane of each panel) on which each kinase or substrate was analyzed in duplicates. The experiment and assay was performed twice. The positions of β-catenin (green box), pGsk3β (red box), and CREB (blue box) are highlighted. (**c**) Western blot analysis showed the phosphorylation of Gsk3β and indicated a slightly enhanced β-catenin expression in SW620 cells stimulated with SEA and HEK-IPSE for 4 h. Gapdh was used as loading control. The experiment and assay were reproduced at least two times. Representative blots are shown. The quantification is presented in Suppl. Fig. 1. (**d**) Inhibition of Wnt-signaling by the tankyrase inhibitor XAV939 demonstrated a reduction of SEA-induced β-catenin. The experiment and assay were reproduced at least two times. The cells were pretreated with XAV939 for 15 h and during the 4-h-stimulation. Representative blots are shown. The quantification is presented in Suppl. Fig. 2. (**e**) Immunostaining of β-catenin in the colon of control hamsters visualized low amounts of epithelial β-catenin at the luminal side of the colon (black arrowheads), but not in those enterocytes inside the crypts (magnification of the boxed area is depicted in the right panel). (**f**) Enhanced expression of β-catenin was observed at the bottom of those crypts around the extravasation sites of *S. mansoni* eggs in bisex-infected hamster colon (red arrows). Please note that enhanced nuclear translocation of β-catenin in enterocytes at the bottom of those crypts, which surround the extravasation sites of *S. mansoni* eggs in bisex-infected hamster colon (red arrowheads in the magnified area, right panel). The black arrows depict enhanced intercellular accumulation of β-catenin. *S. mansoni* eggs (*), magnification 200×, bar 200 µm (left panels) and 1000×, bar 20 µm (right panels). The quantification is presented in Suppl. Fig. 3.

To date, the effects of *S. mansoni* egg-induced colitis on enterocytes remain poorly understood. Therefore, we performed a phospho-kinase array to detect possible impact of *S. mansoni* egg-secreted antigens on distinct intracellular signaling pathways in colon epithelial cells. The Human Phospho-Kinase Array is a rapid and sensitive tool to simultaneously detect the relative levels of phosphorylation of kinases and two related total proteins, which is essential for understanding how cells recognize and respond to changes in their environment. cAMP-response element binding protein (CREB S133), AMP-dependent Kinase (AMPKa1 T183), Heat shock protein 27 (Hsp27 S78/S82), Epidermal Growth Factor Receptor (EGFR Y1086), Gsk3β S21/S9, β-catenin (unphosphorylated), and other factors were induced by stimulation of the human epithelial cell line SW620 with SEA (Fig. 1a,b). Given its role as a potential therapeutic target in CRC, we further focused on the Wnt/β-catenin pathway. Both SEA and the stimulation of SW620 with raising concentrations of HEK-IPSE induced the phosphorylation of the inhibitory phosphorylation site Ser9 of Gsk3β (Fig. 1c, quantification in Suppl. Fig. 1). While we observed a trend of β-catenin expression following SEA stimulation (Fig. 1c, Suppl. Fig. 1), the inhibition of Wnt-signaling by the tankyrase inhibitor XAV939 led to a clear reduction of β-catenin after SEA stimulation (Fig. 1d, Suppl. Fig. 2).

In order to visualize the cellular activation pattern of the Wnt/β-catenin-signaling pathway in vivo, immunohistochemical stainings of β-catenin were performed on colon sections of *S. mansoni*-infected hamsters. It appears noteworthy that especially enterocytes in crypts adjacent to extravasating eggs stained positive for β-catenin (Fig. 1e). Higher magnifications of β-catenin-positive crypts revealed both an enhanced cytoplasmatic and nuclear staining (Fig. 1f, quantification in Suppl. Fig. 3). Taken together, these results suggest that *S. mansoni* egg-secreted factors could activate Wnt-signaling in colonic enterocytes in direct proximity to extravasation sites of *S. mansoni* eggs.

### *Schistosoma mansoni* infection provoked fibrogranulomatous colitis in patients and hamsters

To assure life cycle progression, *S. mansoni* eggs need to egress from the human body. Arising in the mesenteric veins, *S. mansoni* eggs pass through the intestinal wall into the gut lumen^10^*.* The histological examination of human colon biopsies showed multiple parasite eggs along with pronounced multifocal granulomatous inflammation in the mucosa and submucosa (Suppl. Fig. 4a,b). Colon samples of *S. mansoni*-infected hamsters presented a comparable phenotype (Suppl. Fig. 4c–f). The submucosa appeared thickened at the sites of egg-extravasation, and polyp-like structures were identified (dashed line, Suppl. Fig. 4c,e). Granulomatous inflammation was accompanied by marked fibrosis, which was visualized by Masson’s trichrome staining (arrowheads, Suppl. Fig. 4e,f). In conclusion, the hamster model for *S. mansoni* infection used in this study demonstrated typical histological features of *S. mansoni*-induced colitis, comparable to those found in humans^2,10^.

### The protooncogene c-Jun was activated in enterocytes at extravasation sites of *S. mansoni* eggs

**Figure 2:**
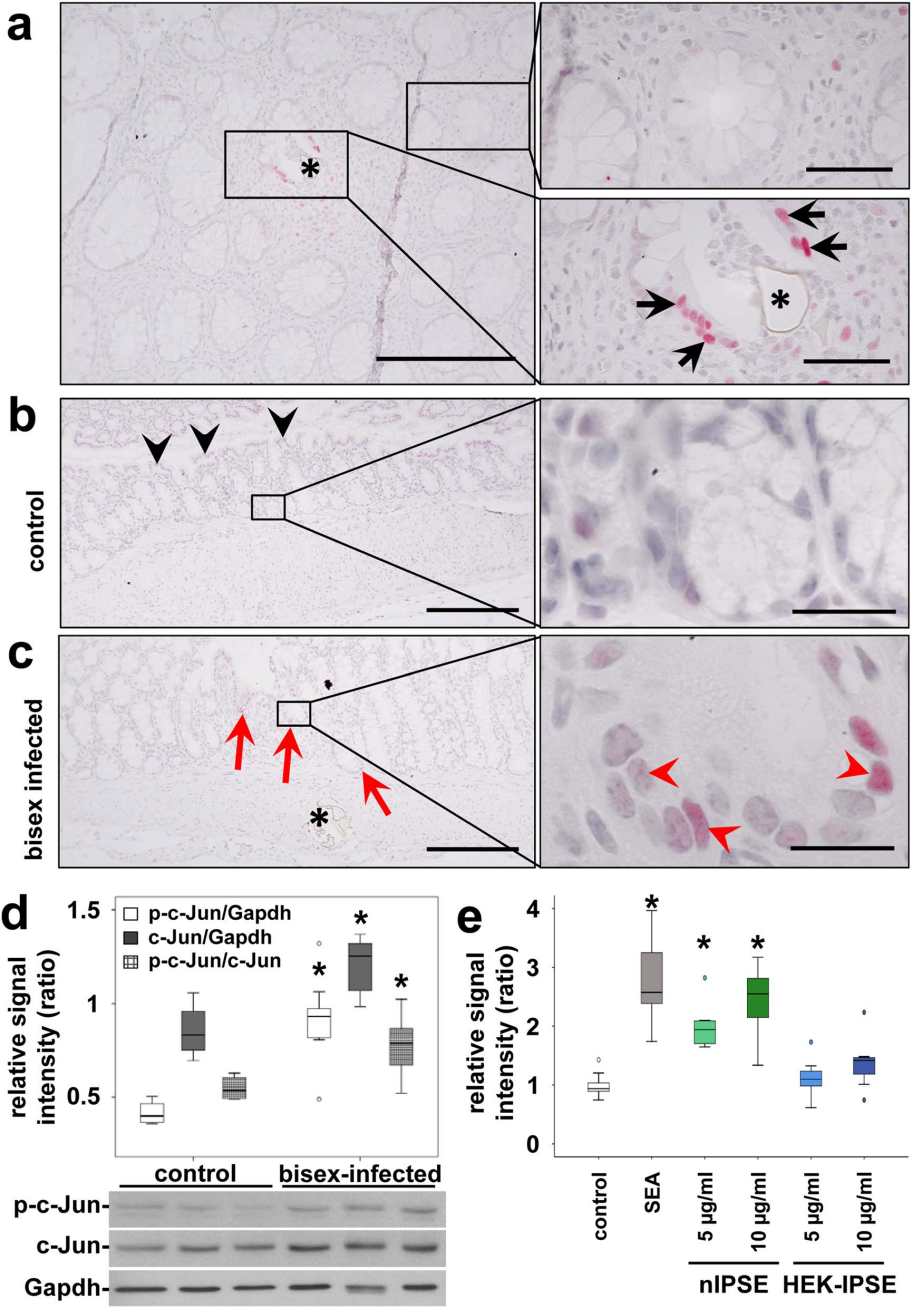
Activation of the protooncogene c-Jun in enterocytes at extravasation sites of *S. mansoni* eggs. (**a**) A representative histologic slice of a rectal biopsy from an 29 year-old Egyptian male with schistosomal colitis is shown. Immunohistochemical staining of c-Jun (red) depicted its nuclear translocation (arrows) in enterocytes of crypts in direct vicinity of *S. mansoni* eggs (*, lower right panel). Nearly no nuclear translocation of c-Jun was observed in enterocytes lining unaffected crypts at a distance of at least 200 µm (upper right panel). The same result was shown in at least three samples from different patients. Co-staining of nuclei in blue, magnification 200× and 1000×, bars 200 µm (left panel) and 50 µm (panel on the right). (**b**) Immunostaining visualized low amounts of epithelial c-Jun at the luminal side of the colon (black arrowheads), but not in enterocytes inside the crypts (magnification of the boxed area is depicted in the right panel). (**c**) Immunostaining demonstrated enhanced nuclear translocation of c-Jun (red arrowheads) in enterocytes of crypts (red arrows) adjacent to sites of *S. mansoni* egg (*) extravasation in bisex-infected hamsters. The quantification is presented in Suppl. Fig. 5. Magnified representative crypts are shown on the right. Magnification in (**b**,**c**) 200× and 1000×, bars 200 µm and 20 µm. (**d**) Western blot analysis and subsequent semi-quantitative assessment of optical density suggested an enhanced expression and activation of c-Jun (p-Ser73) in the colon of *S. mansoni* bisex-infected hamsters in comparison to single sex-infected hamsters, lacking egg production. The experiment and assay were reproduced at least two times. Representative blots are shown, control n = 4, bisex infection n = 11. Differences were analyzed statistically between control and bisex-infected for the activated form (p–c-Jun), total expression (c-Jun), and the ratio between p–c-Jun and c-Jun. *p ≤ 0.05. (**e**) The reporter gene assay demonstrated, that SEA and IPSE stimulation lead to the functional activation of the AP1 promotor. The experiment and assay were reproduced at least two times and analyzed by Kruskall-Wallis test. *p ≤ 0.05.

We recently reported the constitutive activation of the hepatocellular carcinoma-associated protooncogene c-Jun in hepatocytes by factors released from tissue-trapped *S. mansoni* eggs^21^. Immunohistochemical analyses of colon samples derived from both *S. mansoni*-infected patients and our hamster model yielded similar results, indicating the nuclear translocation of c-Jun in the enterocytes that surround parasite eggs (Fig. 2a–c, Suppl. Fig. 5). In addition to the enhanced nuclear translocation of c-Jun in the enterocytes at the sites of egg extravasation (Suppl. Fig. 5), the expression, and phosphorylation of c-Jun was globally enhanced in the colon of bisex-infected animals (Fig. 2d).

Interestingly, we detected a comparable, but much weaker activation pattern of c-Jun in the small bowel of *S. mansoni*-infected hamsters (Suppl. Fig. 6). However, nuclear translocation of c-Jun was less prominent in the small bowel, and the assessment of protein expression and activation by western blot yielded no differences in ileal c-Jun activation between bisex-infected hamsters and controls.

### SEA and IPSE lead to c-Jun activation in vitro

**Figure 3:**
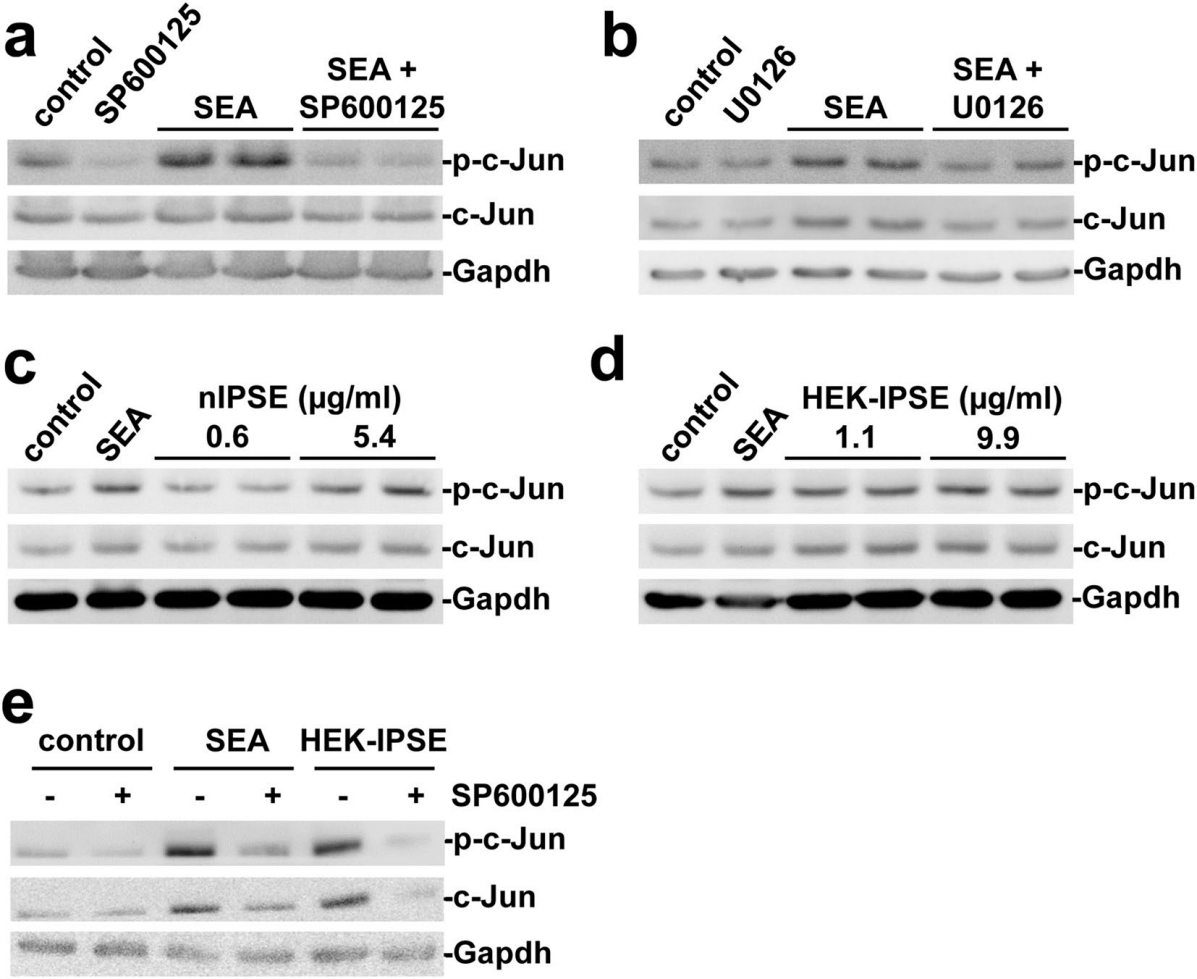
SEA and IPSE activated c-Jun in vitro*.* (**a**,**b**) The expression and phosphorylation of c-Jun increased following SEA-stimulation and was inhibited by additional treatment with the JNK-inhibitor SP600125 (**a**) and the MEK-inhibitor U0126 (**b**) in SW620 cells. (**c**,**d**) A concentration-dependent induction of expression and activation of c-Jun by nIPSE (**c**) and HEK-IPSE (**d**) was shown by western blot. (**e**) SEA- and HEK-IPSE-induced activation of c-Jun was inhibited by the JNK-inhibitor SP600125. The experiment and assay were reproduced at least two times. Representative blots are shown. The cells were pretreated with inhibitors for 30 min and subsequently for 4 h during stimulation.

Our results suggested a mechanism of c-Jun activation that depends on substances released from *S. mansoni* eggs. To further substantiate these findings, we next performed in vitro experiments, again utilizing human SW620 cells. A reporter gene assay demonstrated, that SEA and IPSE stimulation lead to the functional activation of the AP1 promotor (Fig. 2e). The induction of c-Jun protein expression and phosphorylation in consequence of the SEA stimulation was demonstrated by western blot (Fig. 3a,b). Moreover, the activation of c-Jun was attenuated using a selective and cell permeable inhibitor of c-Jun N-terminal kinase (JNK, SP600125) and the MEK1/2-inhibitor U0126 (Fig. 3a,b). To assess, whether secreted products from *S. mansoni* eggs were responsible for the activation of c-Jun, we repeated the experiments with natural IPSE (nIPSE), purified from *S. mansoni* eggs, and recombinant IPSE produced from HEK cells (HEK-IPSE). Western blot analysis revealed a concentration-dependent activation of c-Jun (Fig. 3c,d) which was reversed by the addition of SP600125 (Fig. 3e).

### *Schistosoma mansoni* eggs induced the expression of proliferation markers

**Figure 4:**
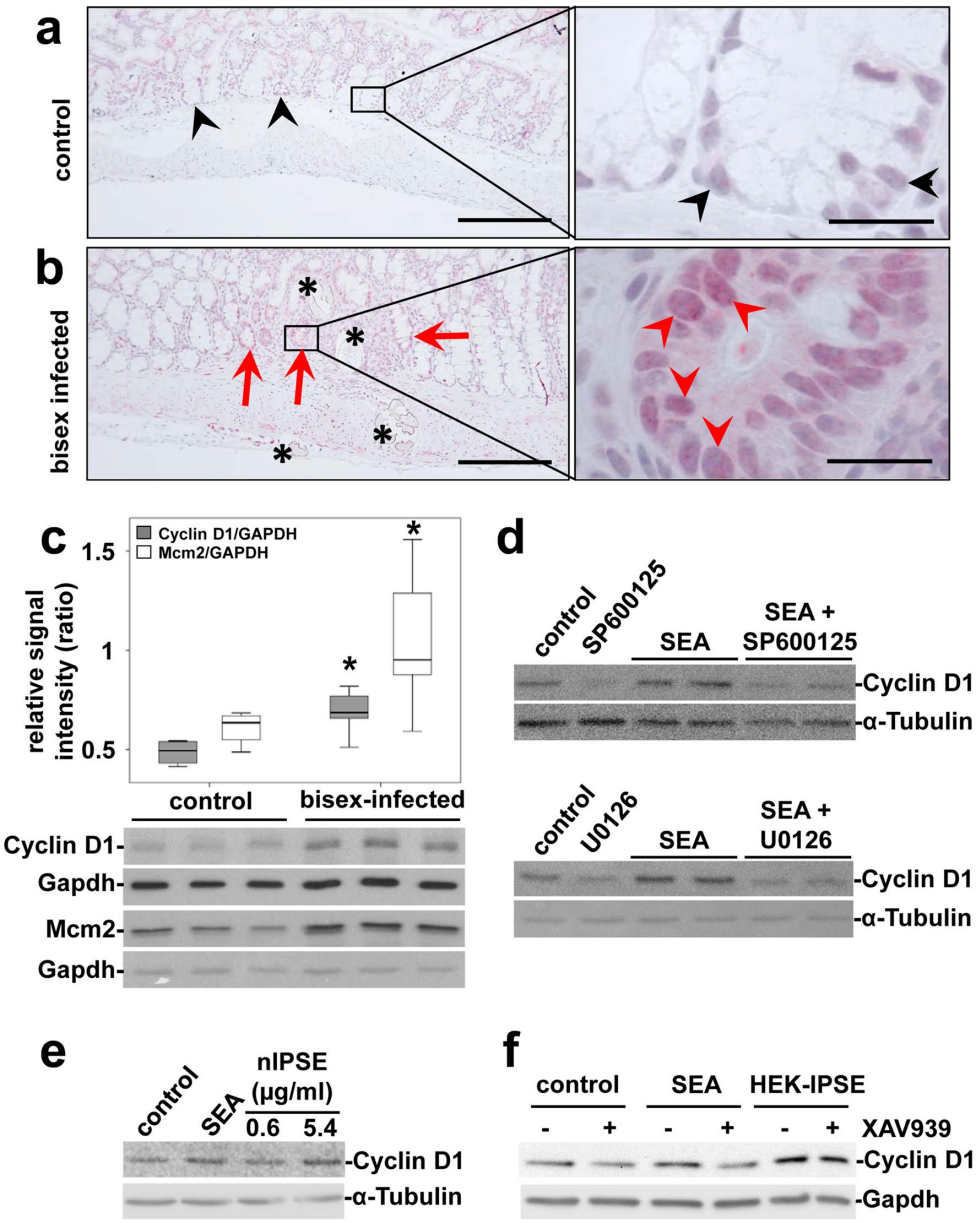
*S. mansoni* egg-induced expression of proliferation markers. (**a**,**b**) Increased enterocyte proliferation, denoted by Cyclin D1-immunostaining adjacent [red arrowheads in (**b**)] to the eggs (*) was distinct from physiological proliferation of enterocytes in basal crypts [black arrowheads in (**a**)]. Representative cross sections of colon mucosa from hamsters are shown.(**a**) single sex-infected control, (**b**) bisex-infected. Magnification 200×, bar 2200 µm and 1000×, bar 20 µm. (**c**) Western blot analysis and subsequent semi-quantitative assessment of optical density suggested an enhanced expression of Cyclin D1 and Mcm2 in the colon of *S. mansoni* bisex-infected hamsters in comparison to single sex-infected controls. Representative blots are shown, control n = 4, bisex infection n = 11. Differences were analyzed statistically between control and bisex-infected for Cyclin D1/Gapdh and Mcm2/Gapdh. *p ≤ 0.05. (**d**) The expression of Cyclin D1 was induced by SEA and could be inhibited by additional treatment with the JNK-inhibitor SP600125 (upper blots) and the MEK-inhibitor U0126 (lower blots) in SW620 cells. The cells were pretreated with inhibitors for 30 min and subsequently during stimulation for 4 h. (**e**) The expression of Cyclin D1 was also induced by nIPSE. (**f**) SEA-stimulated Cyclin D1 expression was reduced by tankyrase inhibition, using XAV939, in SW620 cells. IPSE-induced Cyclin D1 expression, however, was not reduced by tankyrase inhibition. The cells were pretreated with XAV939 for 15 h and subsequently during stimulation for 4 h. The experiment and assay was reproduced at least two times. Representative western blots are shown.

The Wnt-pathway and the transcription factor c-Jun are well characterized key regulators of epithelial cell proliferation in carcinogenesis^36,37^. To monitor proliferation, we analyzed the expression patterns of the cellular proliferation markers Mcm2, Cyclin D1, and Ki67, which have been described as prognostic markers in the context of CRC^38,39^. Enhanced nuclear staining of Cyclin D1 and Ki67 adjacent to *S. mansoni* eggs indicated increased enterocyte proliferation in human (Suppl. Fig. 7) and hamster colon samples (Fig. 4a,b, Suppl. Fig. 8). Immunostaining of Cyclin D1 in cross sections, displaying the distinct layers of the colon mucosa, demonstrated that the enhanced staining surrounding the eggs (Fig. 4b) differentiates from the staining pattern in unaffected areas, resulting from physiological enterocyte proliferation in basal crypts (Fig. 4a). The global Cyclin D1 and Mcm2 expression was increased in the colon of bisex-infected hamsters (Fig. 4c). Furthermore, SEA were capable of inducing Cyclin D1 expression in SW620 cells, which was inhibited by SP600125 and U0126 (Fig. 4d). Also HEK-IPSE induced expression of Cyclin D1 in SW620 cells was inhibited by SP600125 (Suppl. Fig. 9). This indicated that Cyclin D1 induction is a downstream event of JNK- and MEK1/2-activation. Additionally, IPSE stimulation led to Cyclin D1 expression in a concentration-dependent manner (Fig. 4e). The inhibition of Wnt-signaling by XAV939 reduced the SEA- but not the IPSE-induced expression of Cyclin D1 (Fig. 4f).

### *Schistosoma mansoni* eggs induced the expression of markers for DNA repair

**Figure 5:**
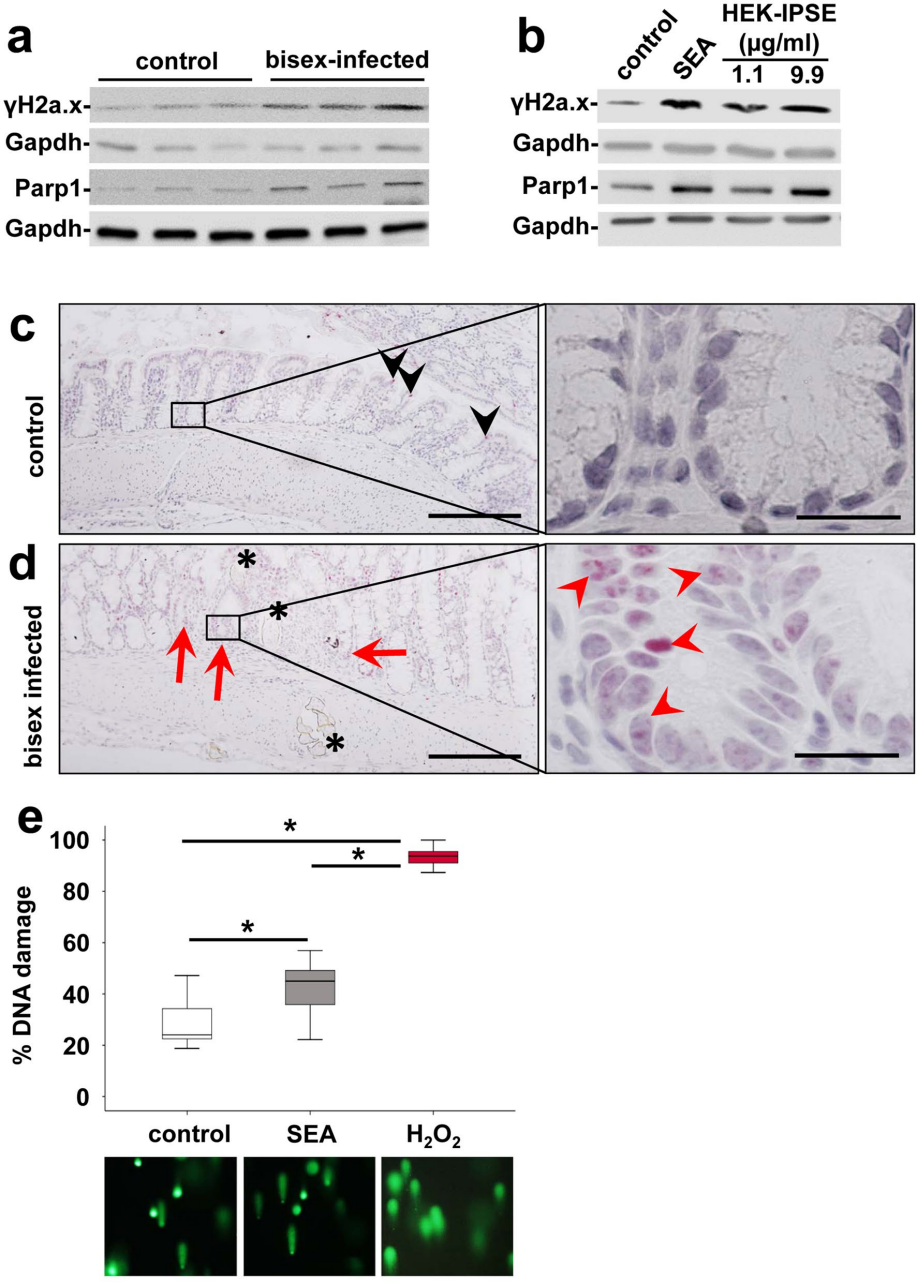
*S. mansoni* egg-induced DNA repair. (**a**) Western blot analysis demonstrated enhanced expression of γH2a.x and Parp-1 in the colon of *S. mansoni* bisex-infected hamsters in comparison to single sex-infected controls. Representative blots are shown. (**b**) Induction of expression and activation of γH2a.x and Parp1 by SEA and HEK-IPSE is shown by western blot (stimulation 4 h). (**c**,**d**) Immunostaining of γH2a.x (blue) depicted nuclear staining (red arrows and arrowheads) in enterocytes of crypts in direct vicinity of *S. mansoni* eggs (*). Representative histologic section of the colon of a control hamster (**c**) and a bisex-infected hamster (**d**) are shown. Quantification of nuclear γH2a.x is demonstrated in Suppl. Fig. 10. Nuclear staining of γH2a.x in red, magnification 200×, bar 200 µm and 1000×, bar 20 µm. (**e**) The comet assay demonstrated enhanced DNA damage by SEA stimulation in SW620 cells. western blot.

Genetic instability and DNA damage are hallmarks of the Fearon and Vogelstein model of colorectal carcinognesis^40^. Hence, we analyzed the expression patterns of specific markers for DNA damage and/or DNA repair, i.e. Parp-1 and γH2a.x. The global expression of γH2a.x and Parp-1 was increased in the colon of bisex-infected hamsters (Fig. 5a). SEA- and HEK-IPSE-treated SW620 cells showed an enhanced expression of γH2a.x and Parp-1 (Fig. 5b). Enhanced nuclear staining of γH2a.x indicated increased DNA double strand breaks in the nuclei of enterocytes adjacent to *S. mansoni* eggs in hamster colon (Fig. 5c,d, Suppl. Fig. 10).

The genotoxic potential of SEA on SW620 cells was analyzed directly by a comet assay (Fig. 5e). In order to quantify DNA damage, the ratio of nuclei with DNA damage (comet tail) in relation to visualized nuclei without DNA damage was assessed in a blinded manner by two persons individually in at least 10 randomly photographed fields of view.

## Discussion

Herein, we provide evidence that *S. mansoni* egg-secreted factors are potent inducers of CRC-associated signaling pathways. Immunostainings suggested the constitutive activation of these factors, apparent in colon epithelial cells in direct proximity to the egg extravasation. Moreover, stimulation- and inhibition-experiments indicated that *S. mansoni* eggs consistently induce prerequisite signaling for epithelial hyperplasia in analogy to the Fearon and Vogelstein concept of colorectal carcinogenesis^40^. The protein concentrations of SEA and IPSE, which induced effects in SW620 cells were in a comparable range of concentration at 1–15 µg/ml. Considering that SEA contains around 1–2% IPSE (personal communication by G. Schramm), the similar activation levels of p-Gsk3β, c-Jun, Cyclin D1, γH2a.x, and Parp1 suggest that other SEA factors may contribute to the observed effects.

It has been proposed that processes like DNA damage, mutagenesis, and oncogene activation could modify parenchymal cell proliferation and survival. These processes enhance carcinogenicity upon infection with pathogens like *Clonorchis*, *Opisthorchis*, and *Schistosoma*^17^. Being one of the key cascades regulating development and stemness, the Wnt-signaling pathway is tightly associated with tumorigenesis, most prominently with CRC^37^. Loss of APC is the main driver of Wnt-signaling in CRC and its pivotal role has been highlighted by recent studies underlining the importance of continuous Wnt-signaling for tumor maintenance^37,41^. Cyclin D1 is a typical downstream target of the Wnt-pathway^42^. The induction of Cyclin D1 is associated with β-catenin expression in colorectal adenocarcinoma^43,44^. Herein, the inhibition experiments demonstrate the reduction of β-catenin and Cyclin D1^45^ by inhibition of the Wnt-pathway, thus providing mechanistic evidence for the stimulation of Wnt-signaling by *S. mansoni* SEA (Fig. 1d, Suppl. Figs. 2 and 4f). Upon infection also the group 1 carcinogen *O. viverrini* activates Wnt/β-catenin signaling pathways in a model of cholangiocarcinogenesis^46^. Interestingly, IPSE-stimulated Cyclin D1-induction is not reduced by the inhibition of the Wnt-pathway (Fig. 4F). As SEA is a complex mixture of egg-secreted factors, we assume that complex reciprocal effects might be responsible for this discrepancy. Furthermore, the concomitant activation of distinct signaling pathways might exaggerate or mask downstream effects. Since *S. haematobium* IPSE is also an egg-secreted protein, and promoting proliferation of bladder cancer cells and angiogenesis, it is tempting to speculate that similar mechanisms are in play as indicated in the present article^47^. Furthermore, it is noteworthy to underline that the *Clonorchis* and *Opisthorchis* genomes do not encode IPSE, which may suggest that different carcinogenic mechanisms are involved in cholangiocarcinogenesis induced by these liver flukes.

**Figure 6:**
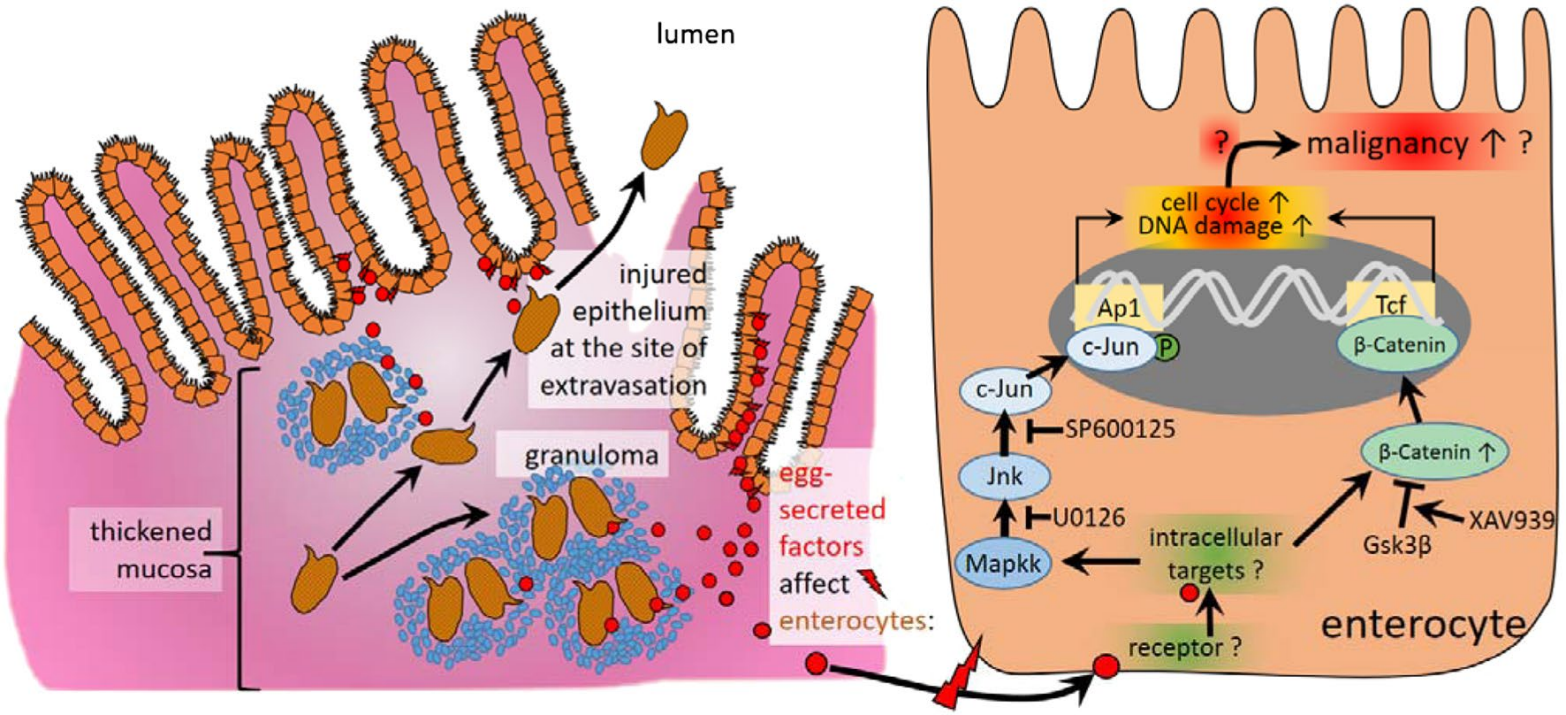
Schematic summary of the main findings of the current study. Granuloma formation around *S. mansoni* eggs trapped within colon tissue is a hallmark of schistosomal colitis. The eggs are metabolically active and highly antigenic. Egg secreted factors (red circles) activate CRC-associated signaling pathways (JNK/c-Jun, Wnt/β-catenin), which initiate proliferation and DNA-damage. These biomolecular processes upon the exposure to schistosomiasis may characterize underlying mechanisms for the predisposition to the development of colorectal cancer as suggested recently^19^.

Our data suggest the induction of two markers for DNA repair, γH2a.x and Parp-1, in the colon of *S. mansoni*-infected hamsters and in SEA- or IPSE stimulated SW620 cells. The induction of phospho-H2a.x indicates DNA double strand breaks and predicts a poor prognosis in CRC^48^. On the other hand Parp-1, which is also causally linked to DNA repair, protects against colorectal tumor induction. But it promotes inflammation-driven colorectal tumor progression^49^. In addition, SEA inducing genotoxic stress has also been demonstrated (Fig. 5e), which strengthens our hypothesis on schistosome eggs inducing DNA damage and genome instability. Taken together our results provide strong evidence, that DNA damage is another egg-induced cancer-promoting mechanism, which refers to the Fearon and Vogelstein concept. The main findings of the current study are summarized in Fig. 6. Precision therapy concepts, focusing on a pharmacologic antagonization of the distinct associated signaling pathways or downstream cellular functions, could be implemented in the therapy of *Schistosoma*-associated CRC. Currently, the control of neglected tropical diseases and the improvement of the basal medical infrastructure in endemic areas, however, remain the primary goal^1^.

It is striking that IBD and other immunopathologies are less prevalent in areas where helminth parasites are endemic^50^. The rising number of studies showing anti-inflammatory effects of helminths, call for the development of translational therapeutic approaches. So far, *S. mansoni* infections, but not egg antigens, have been used to promote recovery from colitis in outbred NMRI mice^51^. Furthermore, it has been shown that helminth-derived compounds induced a Th1/Th2 switch that ameliorated experimental colitis^52,53^. Other studies reported that the coinfection with *S. mansoni* attenuated Crohn’s like inflammatory bowel disease, preserving the epithelial barrier function by downregulating the inflammatory response^54^. In a recently published study, the therapeutic effect of autoclaved *S. mansoni* antigens on toxin induced colon cancer was demonstrated in a murine model^55^. An immunomodulatory effect, characterized by reduced circulatory IL-17 and induced IL-10, as well as the rise of splenic CD4^+^ T-cells and intestinal FoxP3^+^ Treg cells were reported^55^.

Yet, the findings of our study and the aforementioned reports, describing colorectal carcinogenesis in patients with *S. mansoni* infection^19,25,56–58^, should be taken into consideration when designing translational approaches using immunomodulatory properties of helminths and helminth-based vaccination. To this end, it is important to consider our findings in concepts of utilizing immunomodulatory effects of helminth infections as therapeutic approaches in line with the “IBD hygiene hypothesis”^59^. In this context, it should also be reflected that *S. mansoni* infection is associated with alterations to the mammalian intestinal microbiome^60–62^. Finally, detailed insights into the interplay of Th2-prone co-infections or pathologies in the gut and the inflammatory environment established by *S. mansoni* eggs are needed.

In conclusion, we have demonstrated that *S. mansoni* egg-secreted factors induce the Wnt/β-catenin pathway and the protooncogene c-Jun, thus verifying a notable role of *S. mansoni* in intestinal carcinogenesis-associated signaling pathways.

## Supplementary information

Supplementary Information.

## Abbreviations

AP-1: Activator protein 1
CD: Crohn’s disease
CRC: Colorectal carcinoma
CREB: CAMP responsive element binding protein
EMSA: Electrophoretic mobility shift assay
Gapdh: Glyceraldehyde 3-phosphate dehydrogenase
Gsk3β: Glycogen synthase kinase 3
H2A.X: H2A histone family member X
HCC: Hepatocellular carcinoma
HEK-IPSE: Recombinant IPSE produced in human embryonic kidney cells
IBD: Inflammatory bowel disease
IPSE: IL-4-inducing principle from *Schistosoma mansoni* eggs
JNK: C-Jun N-terminal kinase
MCM2: Minichromosome maintenance complex component 2
nIPSE: Natural IPSE, PARP1 Poly [ADP-ribose] polymerase 1
SEA: Soluble egg antigens
SP600125: JNK2 inhibitor
SW 620: Human colon cancer cell line
Th1: T-helper type 1
U0126: MEK1/2-inhibitor
Wnt: Wingless and Int-1
XAV-939: Wnt/β-catenin Inhibitor

## References

[1] Schistosomiasis—fact sheet. https://www.who.int/news-room/fact-sheets/detail/schistosomiasis (2019).

[2] Schwartz C, Fallon PG (2018). *Schistosoma*, "eggs-iting" the host: granuloma formation and egg excretion. Front. Immunol..

[3] Hatz CFR (2005). Schistosomiasis. An underestimated problem in industrialized countries?. J. Travel Med..

[4] Lingscheid T (2017). Schistosomiasis in European travelers and migrants. analysis of 14 years TropNet surveillance data. Am. J. Trop. Med. Hyg..

[5] Boissier J (2016). Outbreak of urogenital schistosomiasis in Corsica (France). An epidemiological case study. Lancet Infect. Dis..

[6] Mulero S, Rey O, Arancibia N, Mas-Coma S, Boissier J (2019). Persistent establishment of a tropical disease in Europe: the preadaptation of schistosomes to overwinter. Parasites Vectors.

[7] Elbaz T, Esmat G (2013). Hepatic and intestinal schistosomiasis: review. J. Adv. Res..

[8] Olveda DU (2014). The chronic enteropathogenic disease schistosomiasis. Int. J. Infect. Dis..

[9] Colley DG, Bustinduy AL, Secor WE, King CH (2014). Human schistosomiasis. Lancet.

[10] Costain AH, MacDonald AS, Smits HH (2018). *Schistosome* egg migration: mechanisms, pathogenesis and host immune responses. Front. Immunol..

[11] Gu K (2017). Clinical diagnostic value of viable *Schistosoma japonicum* eggs detected in host tissues. BMC Infect. Dis..

[12] Mostafa I (1997). *Schistosomal* colonic polyposis. Gastrointest. Endosc..

[13] Barsoum RS, Esmat G, El-Baz T (2013). Human schistosomiasis clinical perspective: review. J. Adv. Res..

[14] Globocan Cancer Facs. Latest Global Cancer Data. Available at https://www.who.int/cancer/PRGlobocanFinal.pdf.

[15] Howley PM (2015). Gordon Wilson lecture: infectious disease causes of cancer: opportunities for prevention and treatment. Trans. Am. Clin. Climatol. Assoc..

[16] de Martel C (2012). Global burden of cancers attributable to infections in 2008: a review and synthetic analysis. Lancet Oncol..

[17] van Tong H, Brindley PJ, Meyer CG, Velavan TP (2017). Parasite infection, carcinogenesis and human malignancy. EBioMedicine.

[18] El-Tonsy MM (2013). *Schistosoma mansoni* infection: Is it a risk factor for development of hepatocellular carcinoma?. Acta Trop..

[19] Madbouly KM (2007). Colorectal cancer in a population with endemic *Schistosoma mansoni*. Is this an at-risk population?. Int. J. Colorectal. Dis..

[20] Palumbo E (2007). Association between schistosomiasis and cancer. Infect. Dis. Clin. Pract..

[21] Roderfeld M (2020). *Schistosoma mansoni* egg secreted antigens activate HCC-associated transcription factors c-Jun and STAT3 in hamster and human hepatocytes. Hepatology (Baltimore, MD).

[22] Wang Z (2020). Comparison of the clinicopathological features and prognoses of patients with schistosomal and nonschistosomal colorectal cancer. Oncol. Lett..

[23] Bye WA (2018). Strategies for detecting colorectal cancer in patients with inflammatory bowel disease: a cochrane systematic review and meta-analysis. Am. J. Gastroenterol..

[24] Nemeth ZH (2017). Crohn's Disease and Ulcerative Colitis show unique cytokine profiles. Cureus.

[25] Zalata KR (2005). p53, Bcl-2 and C-Myc expressions in colorectal carcinoma associated with schistosomiasis in Egypt. Cell. Oncol. Off. J. Int. Soc. Cell. Oncol..

[26] Meyer NH (2015). A crystallin fold in the interleukin-4-inducing principle of *Schistosoma mansoni* eggs (IPSE/α-1) mediates IgE binding for antigen-independent basophil activation. J. Biol. Chem..

[27] Schramm G (2003). Molecular characterization of an interleukin-4-inducing factor from *Schistosoma mansoni* eggs. J. Biol. Chem..

[28] Kaur I, Schramm G, Everts B, Scholzen T, Kindle KB, Beetz C, Montiel-Duarte C, Blindow S, Jones AT, Haas H, Stolnik S, Heery DM, Falcone FH (2011). Interleukin-4-inducing principle from Schistosoma mansoni eggs contains a functional C-terminal nuclear localization signal necessary for nuclear translocation in mammalian cells but not for its uptake. Infect. Immun..

[29] Loeffler DA (2002). Soluble egg antigens from *Schistosoma mansoni* induce angiogenesis-related processes by up-regulating vascular endothelial growth factor in human endothelial cells. J. Infect. Dis..

[30] Grevelding CG (1999). Genomic instability in *Schistosoma mansoni*. Mol. Biochem. Parasitol..

[31] Hempel F (2019). Depletion of bone marrow-derived fibrocytes attenuates TAA-induced liver fibrosis in mice. Cells.

[32] Roderfeld M (2012). Bone marrow transplantation improves hepatic fibrosis in Abcb4^-/-^ mice via Th1 response and matrix metalloproteinase activity. Gut.

[33] Roderfeld M (2010). Bone marrow transplantation demonstrates medullar origin of CD34^+^ fibrocytes and ameliorates hepatic fibrosis in Abcb4^-/-^ mice. Hepatology.

[34] Mann HB, Whitney DR (1947). On a test of whether one of two random variables is stochastically larger than the other. Ann. Math. Stat..

[35] Holm S (1979). A simple sequentially rejective multiple test procedure. Scand. J. Stat..

[36] Sancho R (2009). JNK signalling modulates intestinal homeostasis and tumourigenesis in mice. EMBO J..

[37] Zhan T, Rindtorff N, Boutros M (2017). Wnt signaling in cancer. Oncogene.

[38] Bahnassy AA (2004). Cyclin A and cyclin D1 as significant prognostic markers in colorectal cancer patients. BMC Gastroenterol..

[39] Guzińska-Ustymowicz K, Pryczynicz A, Kemona A, Czyzewska J (2009). Correlation between proliferation markers: PCNA, Ki-67, MCM-2 and antiapoptotic protein Bcl-2 in colorectal cancer. Anticancer Res..

[40] Fearon ER, Vogelstein B (1990). A genetic model for colorectal tumorigenesis. Cell.

[41] Dow LE (2015). Apc restoration promotes cellular differentiation and reestablishes crypt homeostasis in colorectal cancer. Cell.

[42] Slattery ML (2018). Expression of Wnt-signaling pathway genes and their associations with miRNAs in colorectal cancer. Oncotarget.

[43] Sun K (2019). Tre2 (USP6NL) promotes colorectal cancer cell proliferation via Wnt/β-catenin pathway. Cancer Cell Int..

[44] Jang KY (2012). Expression of cyclin D1 is associated with β-catenin expression and correlates with good prognosis in colorectal adenocarcinoma. Transl. Oncol..

[45] Regmi SC (2015). The anti-tumor activity of succinyl macrolactin a is mediated through the β-catenin destruction complex via the suppression of tankyrase and PI3K/Akt. PLoS ONE.

[46] Yothaisong S (2015). Opisthorchis viverrini infection activates the PI3K/AKT/PTEN and Wnt/β-catenin signaling pathways in a cholangiocarcinogenesis model. Asian Pac. J. Cancer Prev..

[47] Mbanefo, E. C. *et al. IPSE, an Abundant Egg-Secreted Protein of the Carcinogenic Helminth Schistosoma haematobium, Promotes Proliferation of Bladder Cancer Cells and Angiogenesis* (2020). 10.1186/s13027-020-00331-6 PMC7578584 33101456

[48] Lee Y-C (2015). High expression of phospho-H2AX predicts a poor prognosis in colorectal cancer. Anticancer Res..

[49] Dörsam B (2018). PARP-1 protects against colorectal tumor induction, but promotes inflammation-driven colorectal tumor progression. Proc. Natl. Acad. Sci. U.S.A..

[50] Varyani F, Fleming JO, Maizels RM (2017). Helminths in the gastrointestinal tract as modulators of immunity and pathology. Am. J. Physiol. Gastrointest. Liver Physiol..

[51] Bodammer P (2011). *Schistosoma mansoni* infection but not egg antigen promotes recovery from colitis in outbred NMRI mice. Dig. Dis. Sci..

[52] Moreels TG (2004). Concurrent infection with *Schistosoma mansoni* attenuates inflammation induced changes in colonic morphology, cytokine levels, and smooth muscle contractility of trinitrobenzene sulphonic acid induced colitis in rats. Gut.

[53] Heylen M (2014). Worm proteins of *Schistosoma mansoni* reduce the severity of experimental chronic colitis in mice by suppressing colonic proinflammatory immune responses. PLoS ONE.

[54] Pêgo B (2019). *Schistosoma mansoni* coinfection attenuates murine *Toxoplasma gondii*-induced Crohn's-like ileitis by preserving the epithelial barrier and downregulating the inflammatory response. Front. Immunol..

[55] Eissa MM, Ismail CA, El-Azzouni MZ, Ghazy AA, Hadi MA (2019). Immuno-therapeutic potential of *Schistosoma mansoni* and *Trichinella spiralis* antigens in a murine model of colon cancer. Investig. New Drugs.

[56] Soliman AS (1997). Colorectal cancer in Egyptian patients under 40 years of age. Int. J. Cancer.

[57] Herman AM (2017). Colorectal cancer in a patient with intestinal schistosomiasis: a case report from Kilimanjaro Christian Medical Center Northern Zone Tanzania. World J. Surg. Oncol..

[58] Salim HOE, Hamid HKS, Mekki SO, Suleiman SH, Ibrahim SZ (2010). Colorectal carcinoma associated with schistosomiasis A possible causal relationship. World J. Surg. Oncol..

[59] Weinstock JV, Elliott DE (2009). Helminths and the IBD hygiene hypothesis. Inflamm. Bowel Dis..

[60] Floudas A (2019). *Schistosoma mansoni* worm infection regulates the intestinal microbiota and susceptibility to colitis. Infect. Immun..

[61] Jenkins TP (2018). *Schistosoma mansoni* infection is associated with quantitative and qualitative modifications of the mammalian intestinal microbiota. Sci. Rep..

[62] Schneeberger PHH (2018). Investigations on the interplays between *Schistosoma mansoni*, praziquantel and the gut microbiome. Parasites Vectors.

