# Supplementary material for: Schistosoma mansoni eggs induce Wnt/β-catenin signaling and activate the protooncogene c-Jun in human and hamster colon
Source: Sci Rep. 2020 Dec 23;10:22373. doi: 10.1038/s41598-020-79450-4 (PMC7758332; doi:10.1038/s41598-020-79450-4)

Schistosoma mansoni eggs induce Wnt/β-catenin signaling and activate the protooncogene c-Jun in human and hamster colon

Short Title: *S. mansoni* activates CRC-signaling

Jakob Weglage[1#], Friederike Wolters[1#], Laura Hehr[1], Jakob Lichtenberger[1], Celina Wulz[1], Felix Hempel[1], Anne Baier[1], Thomas Quack[2], Kernt Köhler[3], Thomas Longerich[4], Gabriele Schramm[5], Karuna Irungbam[1], Heike Mueller[1], Verena von Buelow[1], Annette Tschuschner[1], Margarete Odenthal[6,7], Uta Drebber[6], Maha El Arousy[8], Leandra N. Z. Ramalho[9], Katrin Bankov[10], Peter Wild[10], Jörn Pons-Kühnemann[11], Jonas Tschammer[11], Christoph G. Grevelding[2], Elke Roeb[1#], Martin Roderfeld[1#*]

[1]Department of Gastroenterology, Justus Liebig University, Giessen, Germany

[2]Institute of Parasitology, BFS, Justus Liebig University, Giessen, Germany

[3]Institute of Veterinary Pathology, Justus Liebig University, Giessen, Germany

[4]Translational Gastrointestinal Pathology, Institute of Pathology, University Hospital Heidelberg, Heidelberg, Germany

[5]Experimental Pneumology, Priority Research Area Asthma & Allergy, Research Center Borstel, Borstel, Germany

[6]Institute of Pathology, University of Cologne, Germany

[7]Center of Molecular Medicine (CMMC), University of Cologne, Germany

[8]Department of Parasitology, Faculty of Medicine, Cairo University, Egypt

[9]Department of Pathology, Ribeirão Preto Medical School, University of São Paulo, Brazil

[10]Dr. Senckenberg Institute of Pathology, University Hospital Frankfurt, Frankfurt am Main, Germany

[11]Institute of Medical Informatics, Justus Liebig University, Giessen, Germany

[#] These authors contributed equally

*Corresponding author: Martin Roderfeld, ORCID 0000-0002-3232-1277, Department of Gastroenterology, Justus Liebig University Giessen, Gaffkystr. 11, D-35392 Giessen, Germany. Phone: +49 641 99 42527; Fax: +49 641 99 42333; E-mail: martin.roderfeld@innere.med.uni-giessen.de

Funding: This work was supported by grants from the German Research Foundation (RO3714/4-1), GILEAD (support program Infectiology 2017), LOEWE-Zentrum DRUID-Flex-Funds, and the University Hospital Giessen and Marburg (UKGM).

Suppl. Fig. 1

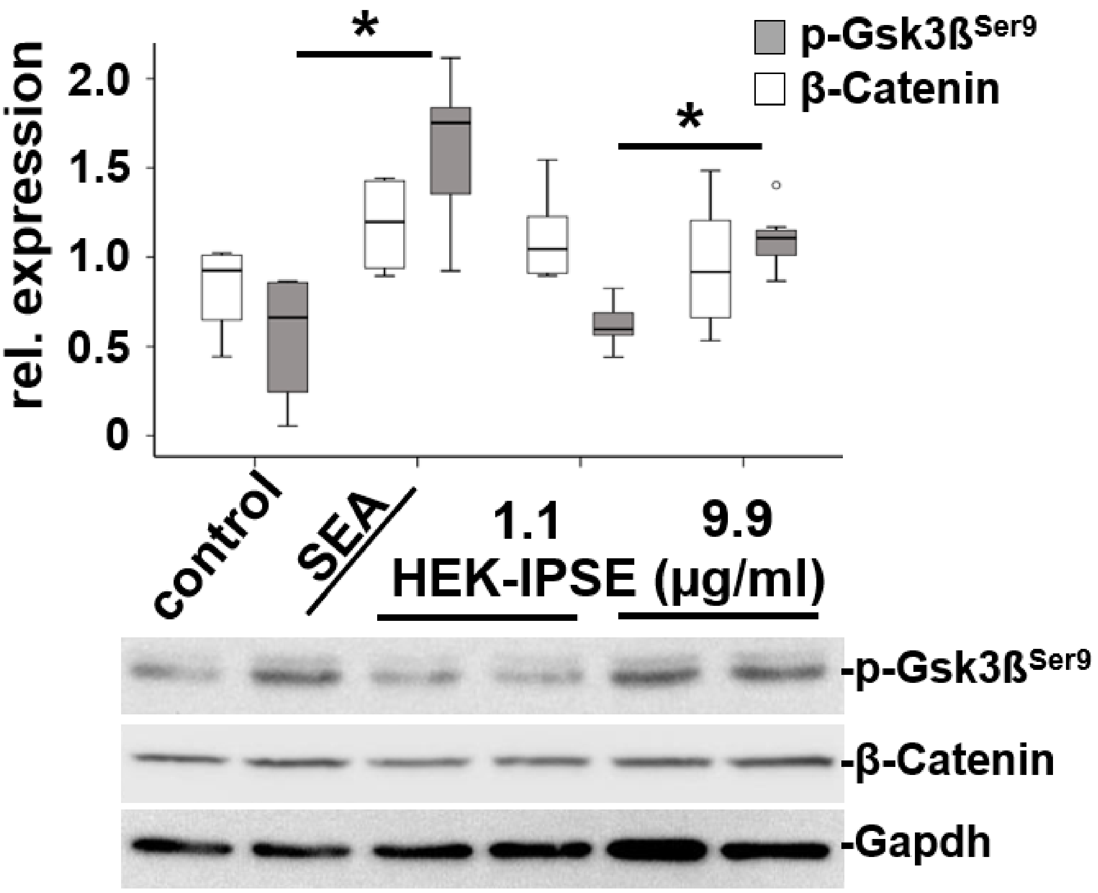

Suppl. Fig. 1: Quantification of western blots shown in Fig. 1c.

Western blot analysis showed the phosphorylation of GSK-3β and indicated a slightly enhanced β-catenin expression in SW620 cells stimulated with SEA and HEK-IPSE for 4h. The level of significance for the difference between the signals for β-catenin in control and HEK-IPSE was lost after Bonferroni correction (p=0.014 before- and p=0.083 after Bonferroni correction). n=3-8/group. GAPDH was used as loading control. The experiment and assay was reproduced at least two times and analyzed by optical densitometry, nonparametric ANOVA (Kruskall-Wallis) and subsequent comparison of the groups as well as Bonferroni correction for statistical analysis. Representative blots are shown.

Suppl. Fig. 2

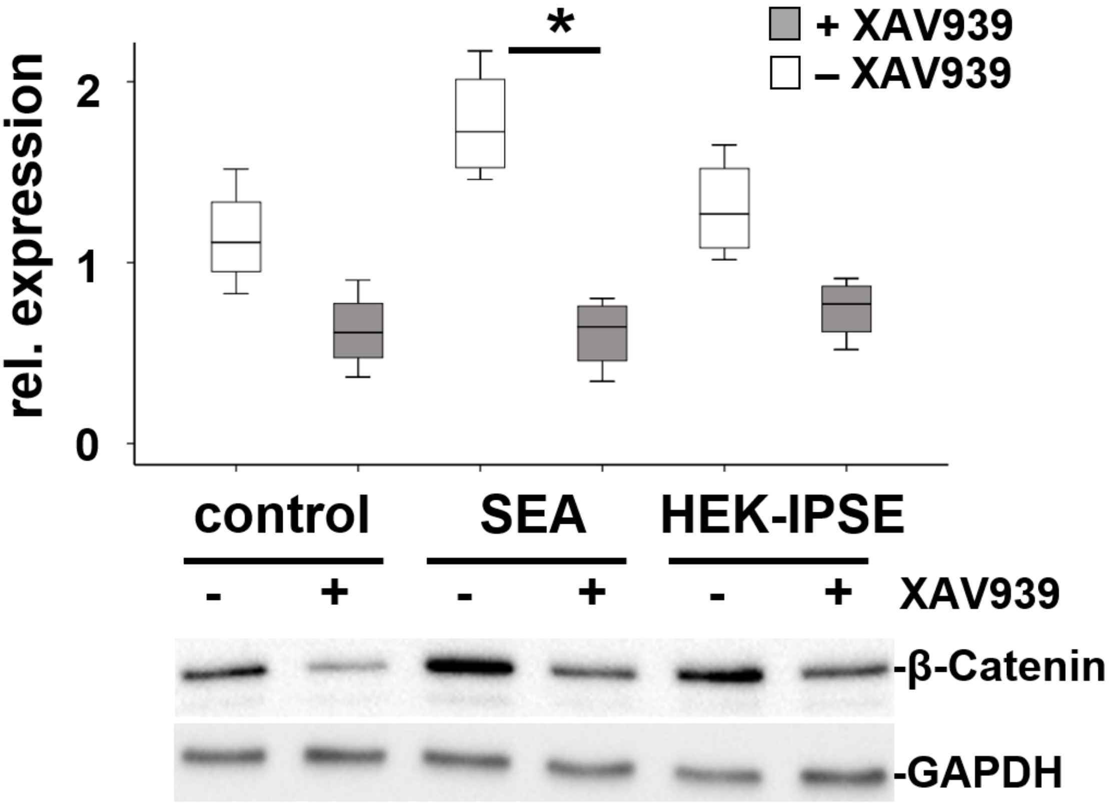

Suppl. Fig. 2: Quantification of western blots shown in Fig. 1d.

The inhibition of Wnt-signaling by the tankyrase inhibitor XAV939 led to a clear reduction of β-catenin after SEA stimulation. n=4/group. GAPDH was used as loading control. The experiment and assay was reproduced at least two times and analyzed by optical densitometry, nonparametric ANOVA (Kruskall-Wallis) and subsequent comparison of the groups as well as Bonferroni correction for statistical analysis. Representative blots are shown.

Suppl. Fig. 3

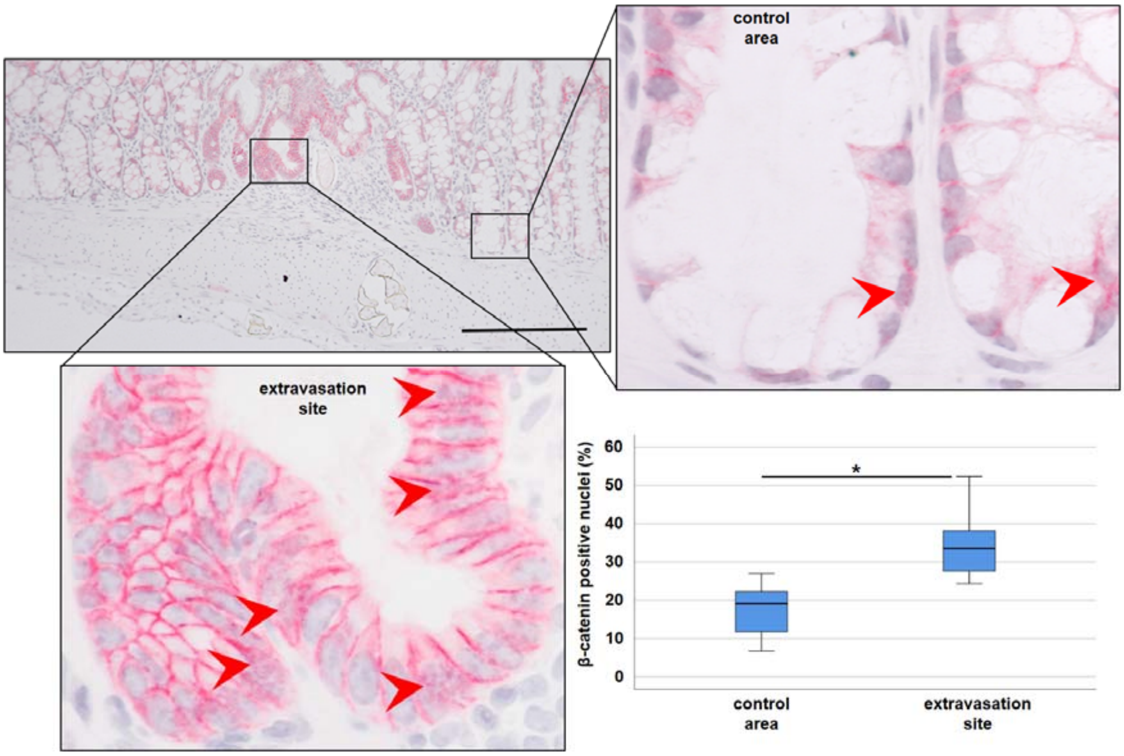

Suppl. Fig. 3: β-catenin positive nuclei of enterocytes in the lower part of cryps in direct vicinity to extravasation sites of *S. mansoni* eggs in bisex infected hamster colon were counted. For control, β-catenin positive nuclei of enterocytes in the lower third of cryps with normal appearance were counted. At least 10 positive and negative area in at least three specimen were used for quantification at 1000x.

Suppl. Fig. 4

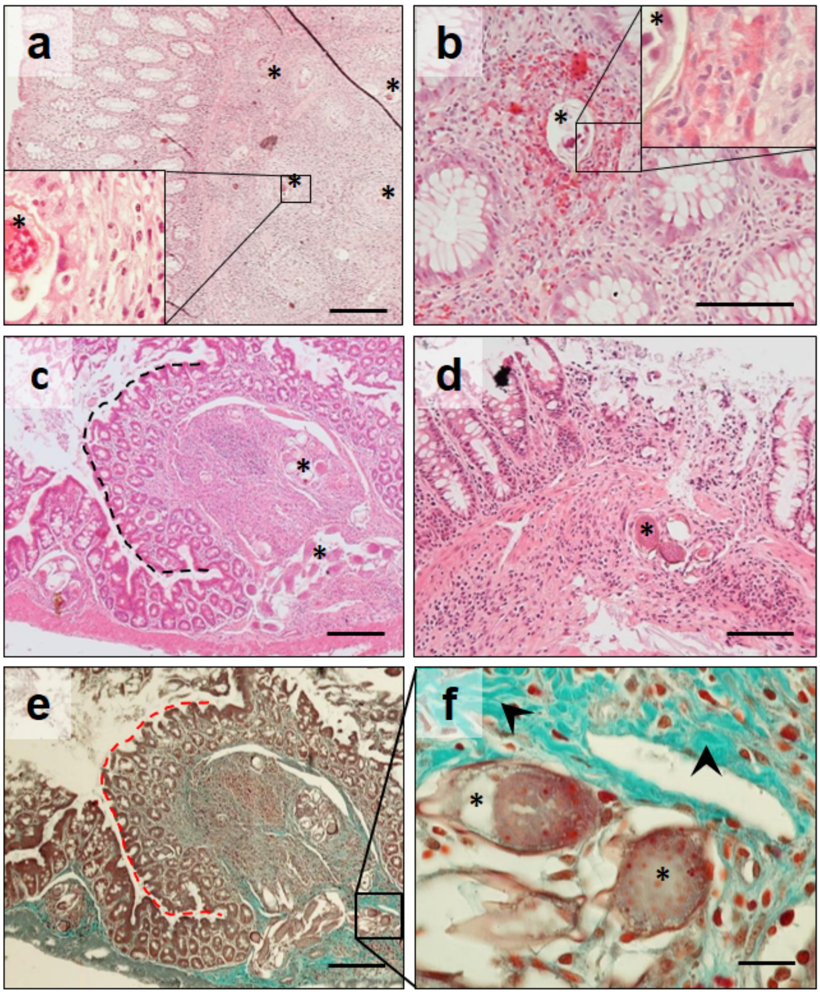

Suppl. Fig. 4: *S. mansoni eggs provoke fibrogranulomatous colitis in patients and infected hamsters*. (A and B) Histologic sections of a rectal biopsy from a 29 year-old male Egypt patient with *S. mansoni*-induced colitis. *S. mansoni* eggs (*) with lateral spines cross the submucosa and mucosa causing a fibroinflammatory reaction with massive infiltration of leucocytes. H.E. staining, magnification 50 x (A) and 200 x (B), bars 200 µm (A) and 100 µm (B). **(C and D)** H.E.-stained histologic sections of *S. mansoni*-infected hamster colon. Pronounced multifocal granulomatous inflammation in the lamina propria with multiple parasitic eggs (*) and distinct edema. Magnification 50 x (C) and 100 x (D), bars 200 µm (A) and 50 µm (B). **(E and F)** Masson's trichrome-stained histologic sections of *S. mansoni*-infected hamster colon. *S. mansoni* eggs (*) passing the bowel wall were surrounded by ~~fibrovascular~~ fibrogranolomatous tissue (arrowheads) leading to ~~granulomatous~~ thickening of the submucosa. (F) shows magnification of the area in the box in (E). Magnification 50 x (E) and 1000 x (F), bars 200 µm (E) and 10 µm (F).

Suppl. Fig. 5

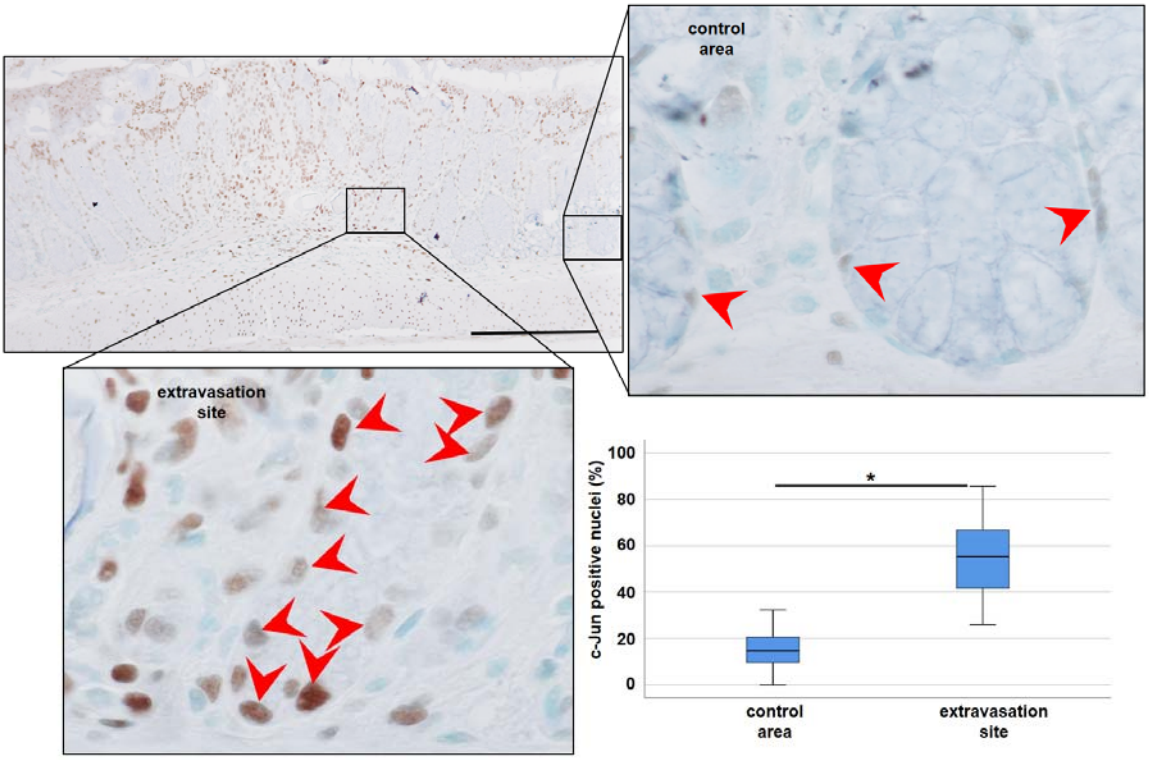

Suppl. Fig. 5: c-Jun positive nuclei of enterocytes in the lower third of cryps in direct vicinity to extravasation sites of *S. mansoni* eggs in bisex infected hamster colon were counted. For control, c-Jun positive nuclei in the lower third of cryps with normal appearance were counted. Red arrowheads depict some of the c-Jun positive nuclei. At least 10 positive and negative area in at least 3 representative specimen were used for quantification at 1000x.

Suppl. Fig. 6

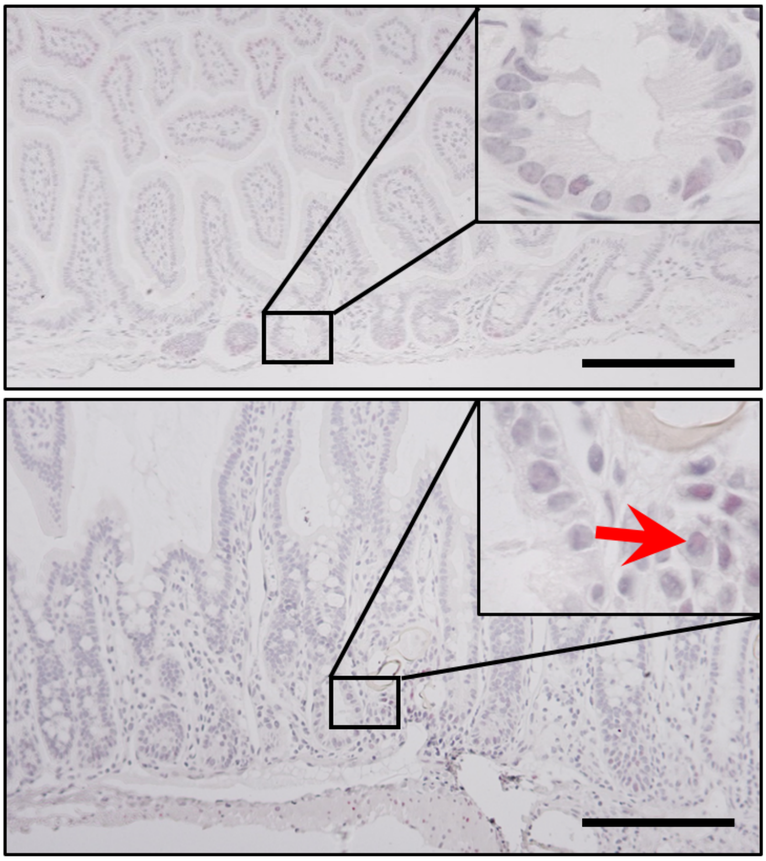

Suppl. Fig. 6: Immunostaining revealed marginal epithelial activation of c-Jun (arrows) around parasite eggs (*) in the ileum of *S. mansoni* infected hamsters (lower panels), which did not differ substantially from c-Jun activation in the ileum of control hamsters (upper panels). Magnification 200 x, bar 200 µm.

Suppl. Fig. 7

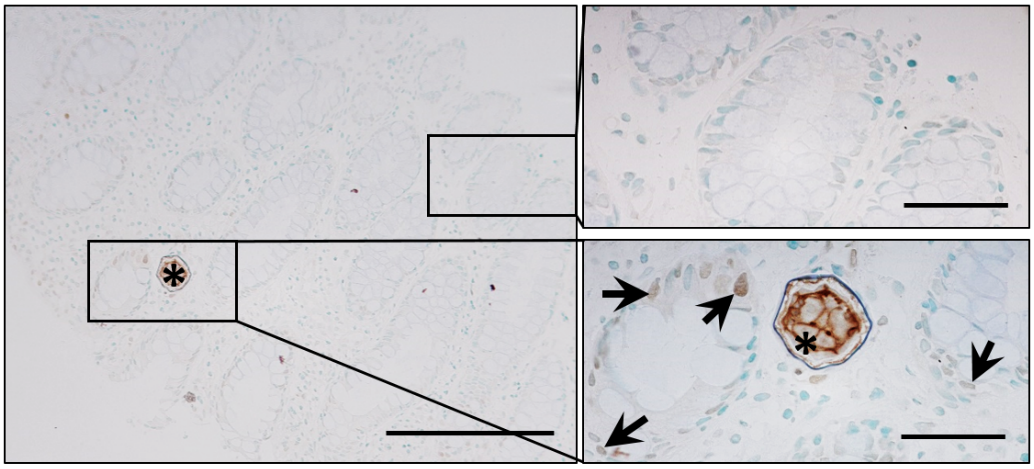

Suppl. Fig. 7: Immunostaining of Cyclin D1 (brown) depicted nuclear localization of Cyclin D1 (arrows) in enterocytes of crypts in direct vicinity to *S. mansoni* eggs (*). The upper panel on the right depicts the magnification of the boxed control area showing normal appearing crypts with marginal Cyclin D1 staining. A representative histologic section of a colon biopsy from an 25-year-old Eritrean male with schistosomal colitis is shown. Nuclear co-staining in blue, magnification 200 x and 1000 x, bars 200 µm and 20 µm.

Suppl. Fig. 8

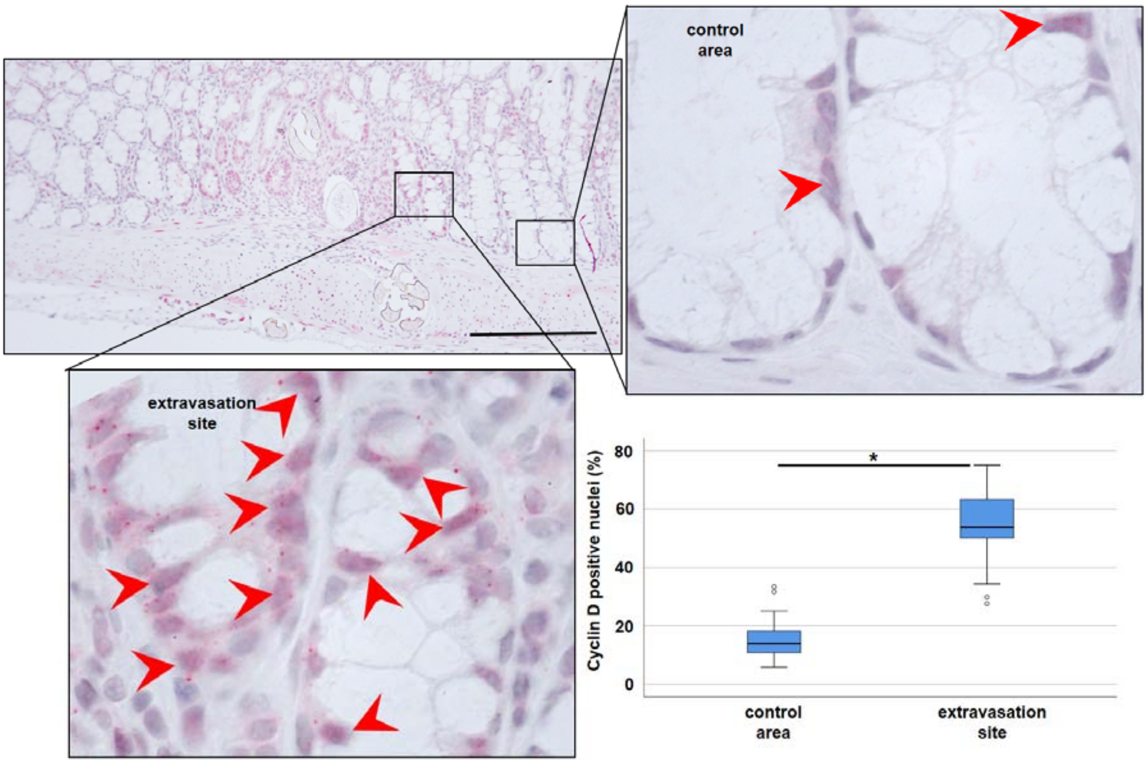

Suppl. Fig. 8: Cyclin D positive nuclei of enterocytes in the lower third of cryps in direct vicinity to extravasation sites of *S. mansoni* eggs in bisex infected hamster colon were counted. For control, Cyclin D positive nuclei of enterocytes in the lower third of cryps with normal appearance were counted. Red arrowheads depict some of the Cyclin D positive nuclei. At least 10 positive and negative area in at least 3 representative specimens were used for quantification at 1000 x.

Suppl. Fig. 9

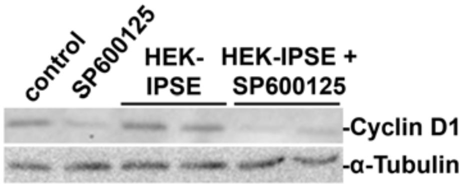

Suppl. Fig. 9: Inhibition of the JNK/c-Jun pathway in SW620 cells by SP600125 reduced SEA activated Cyclin D1 expression. One representative western blot of three.

Suppl. Fig. 10

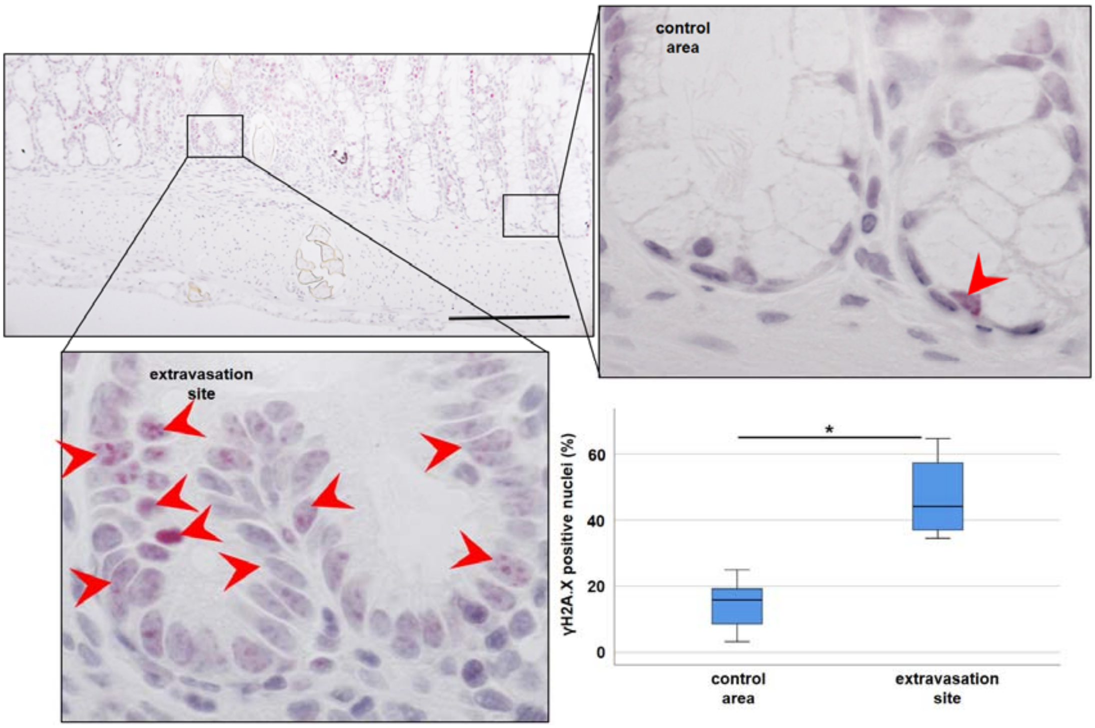

Suppl. Fig. 10: γH2A.X positive nuclei of enterocytes in the lower third of cryps in direct vicinity to extravasation sites of *S. mansoni* eggs in bisex infected hamster colon were counted. For control, γH2A.X positive nuclei of enterocytes in the lower third of cryps with normal appearance were counted. Red arrowheads depict some of the γH2A.X positive nuclei. At least 10 positive and negative area in at least 3 representative specimen were used for quantification.

Original Blots:

Fig. 1a: Array consists of 2 membranes with different analytes for each group.

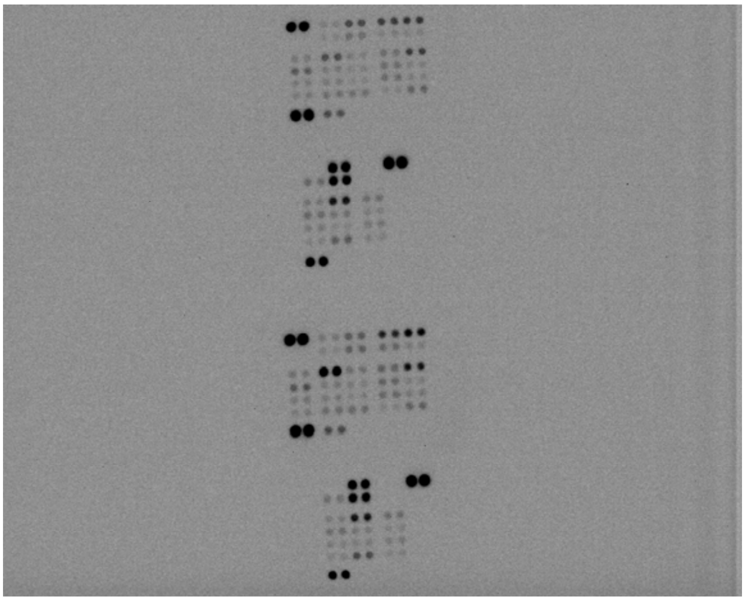

Fig. 1c p-Gsk3ß^{Ser9}: please note, that the standard lane is an incident light photograhy introduced by INTAS imager

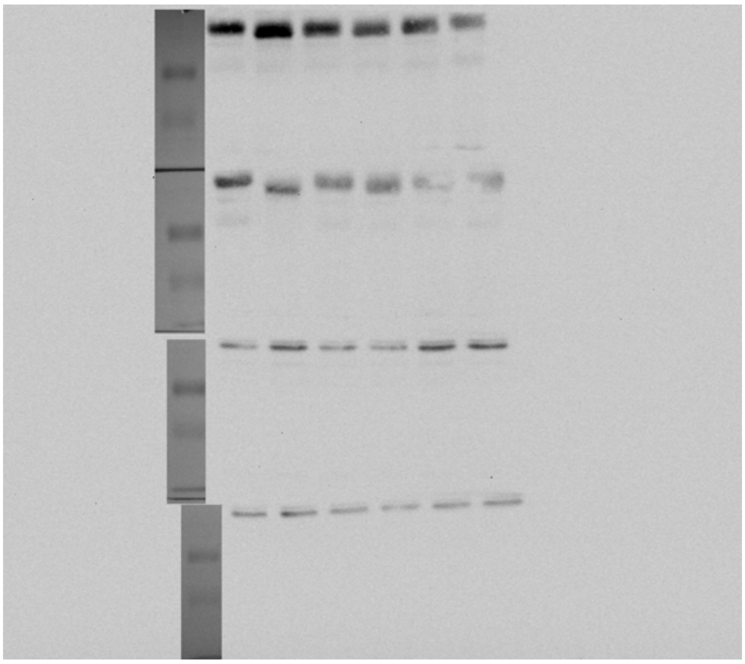

Fig. 1c β-Catenin:

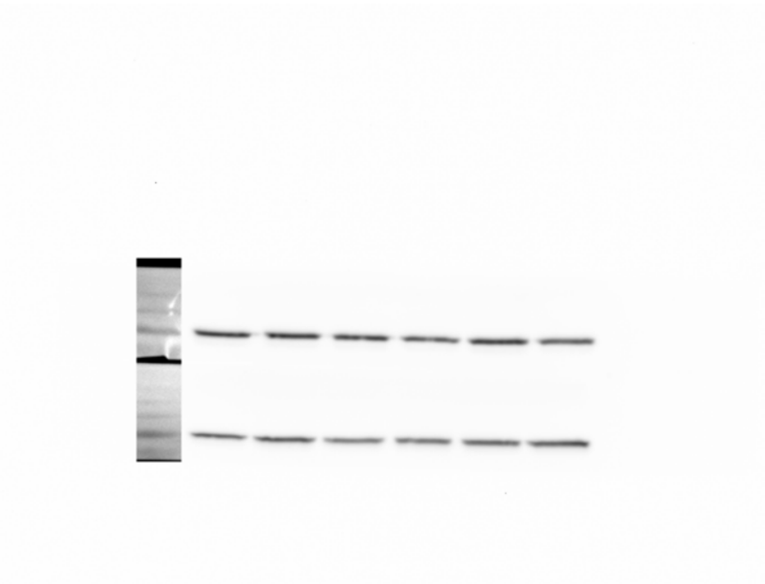

Fig. 1c Gapdh:

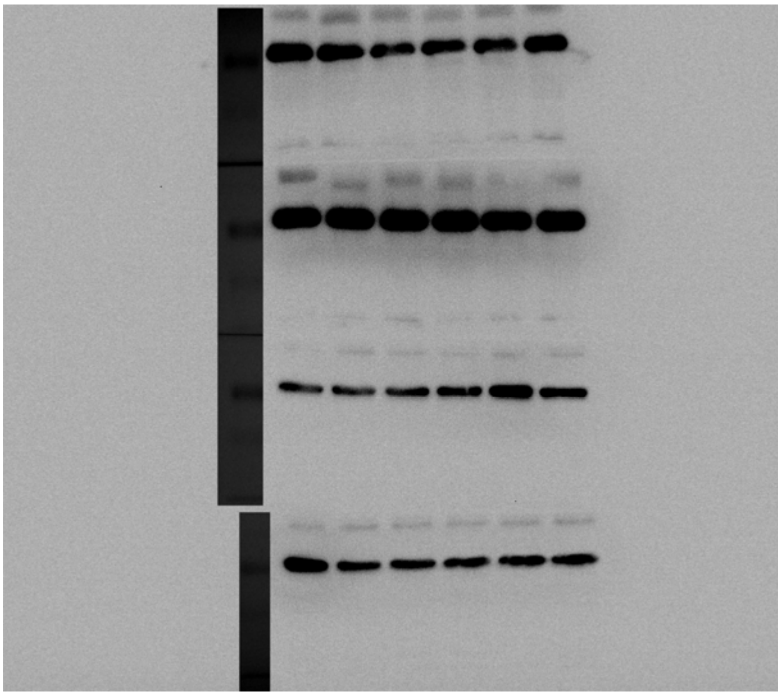

Fig. 1d β-catenin

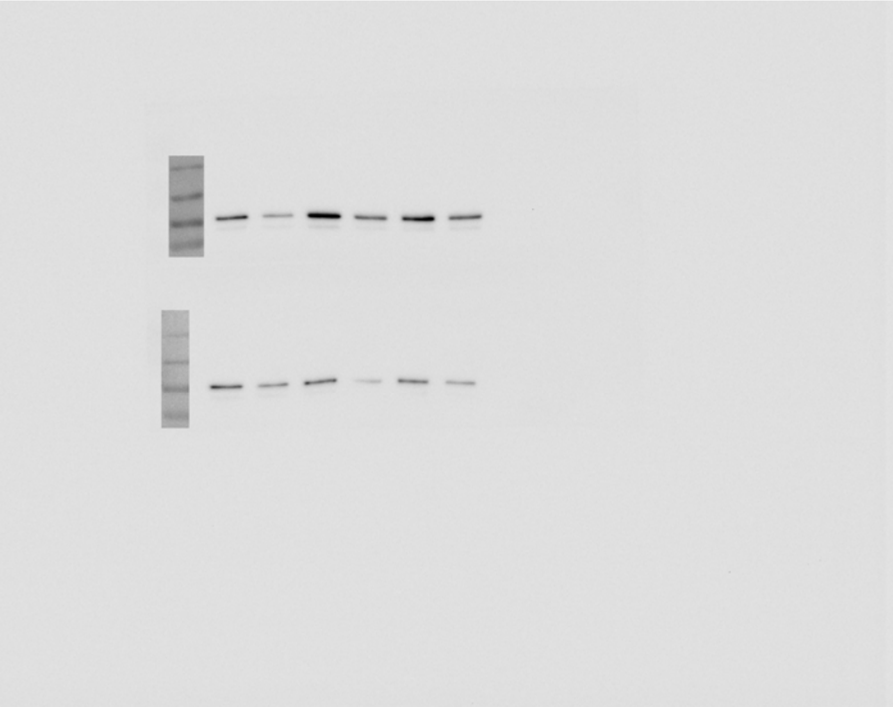

Fig. 1d Gapdh

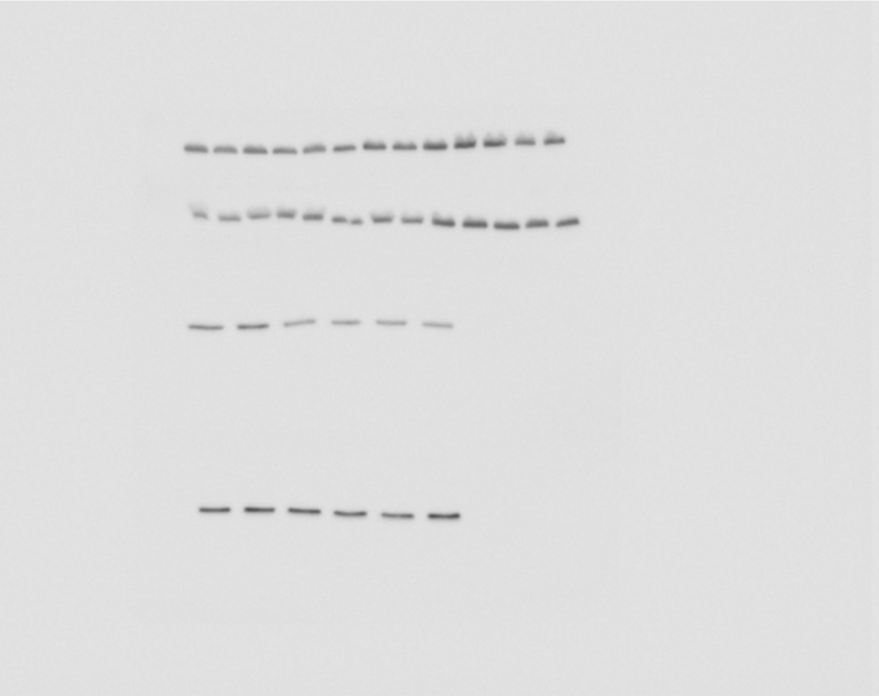

Fig. 1d p-β-Catenin: (old before revision)

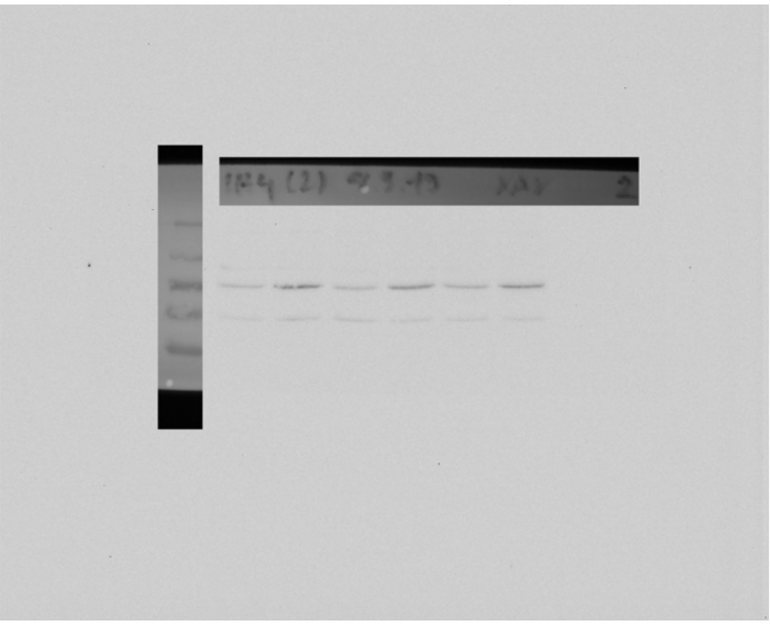

Fig 1d α-Tubulin: (old before revision)

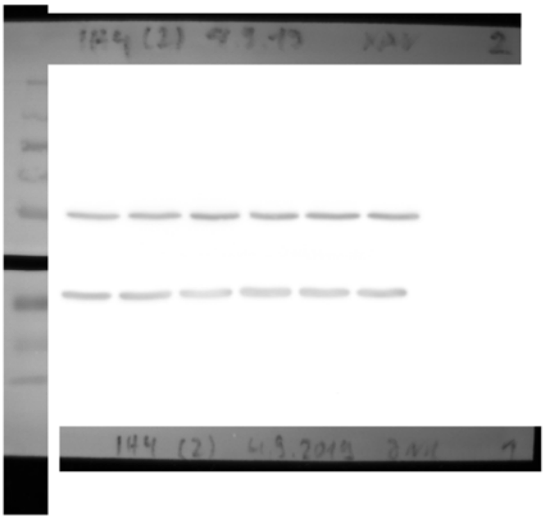

Fig. 2b p-c-Jun:

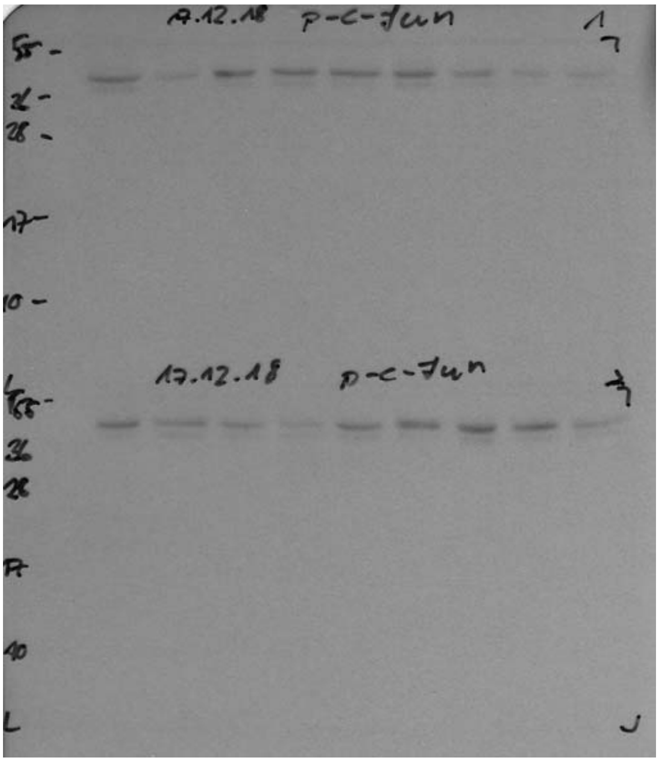

Fig 2b c-Jun:

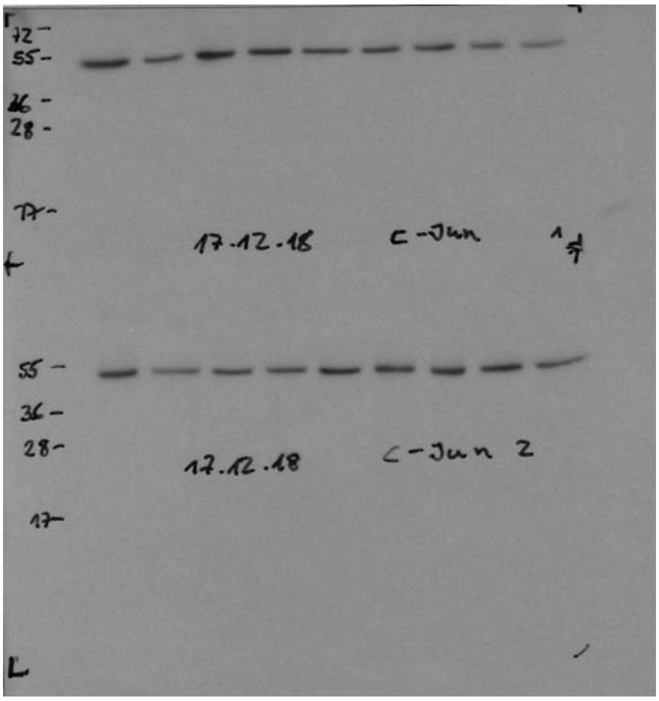

Fig. 2b Gapdh:

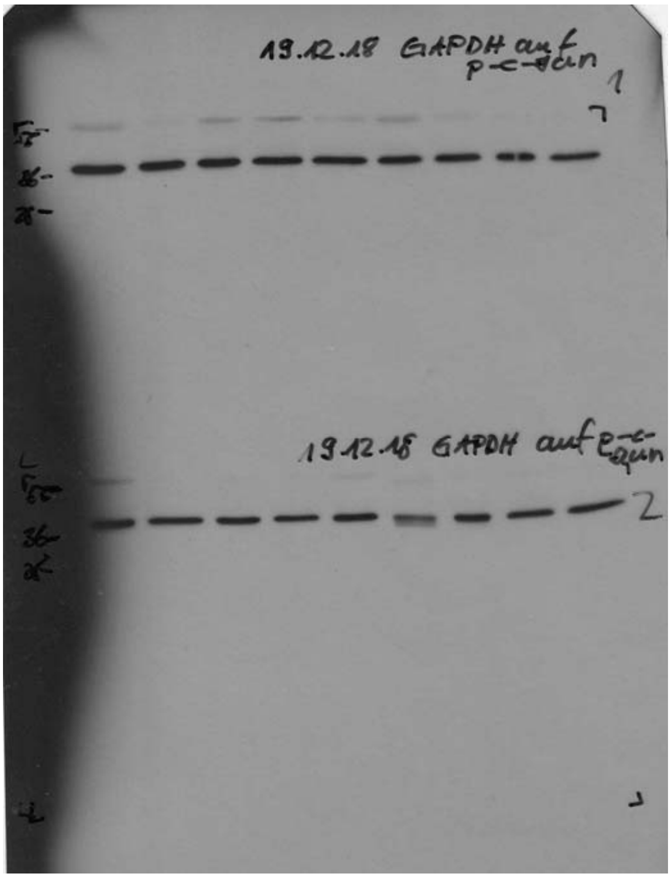

Fig. 2e AP-1 EMSA:

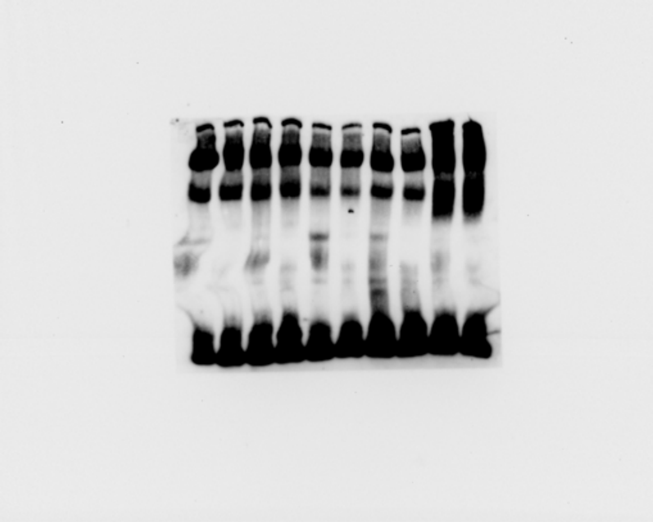

Fig. 3a p-c-Jun:

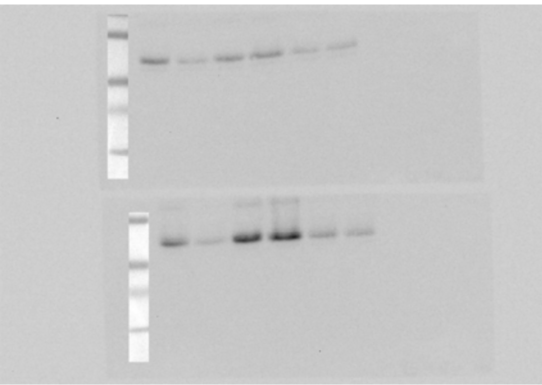

Fig 3a c-Jun:

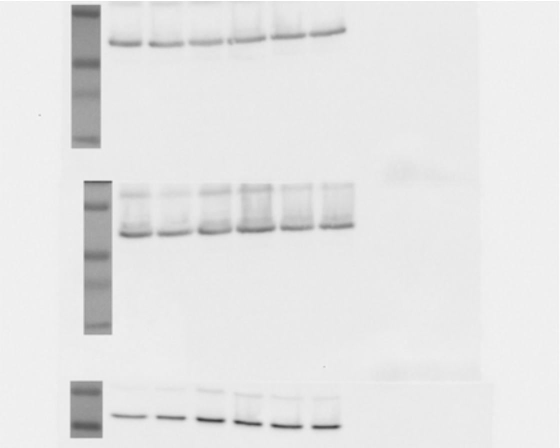

Fig. 3a Gapdh:

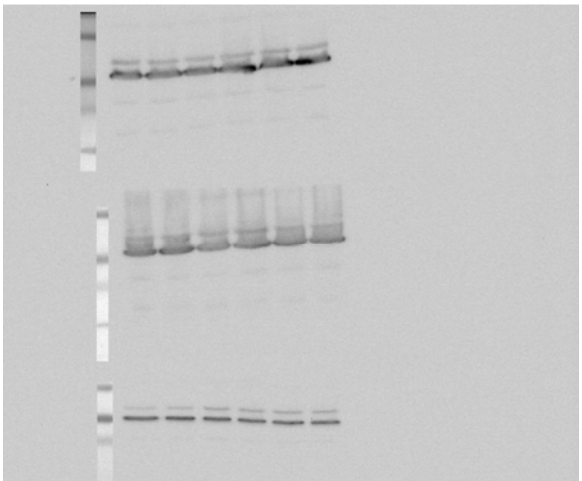

Fig. 3b p-c-Jun:

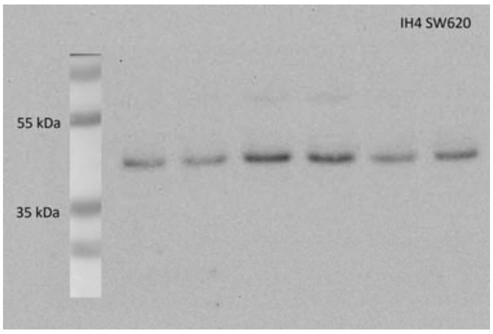

Fig. 3b c-Jun:

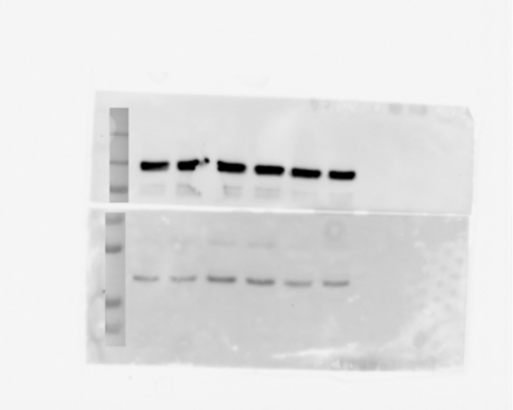

Fig. 3b Gapdh:

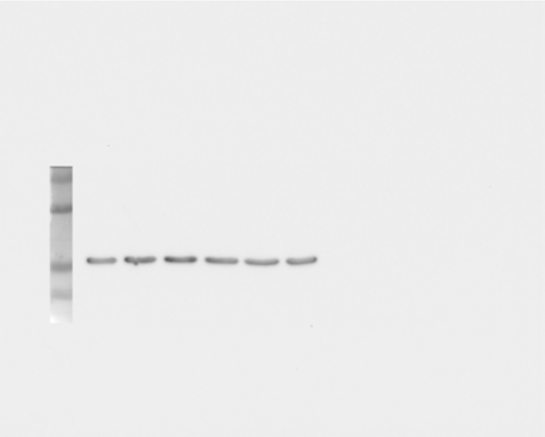

Fig. 3c + d p-c-Jun:

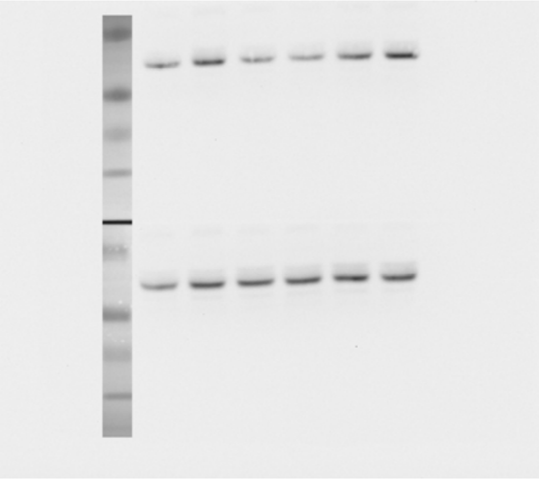

Fig. 3c+d c-Jun:

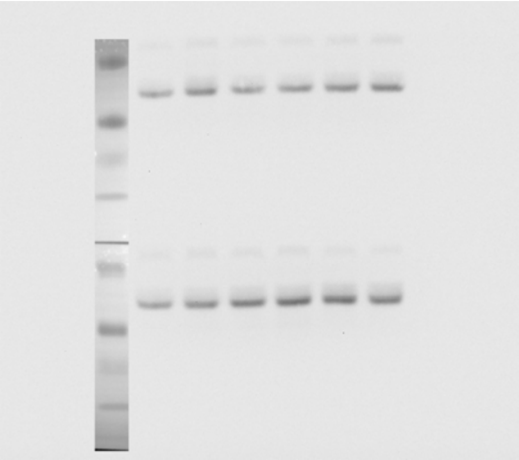

Fig. 3c+d Gapdh:

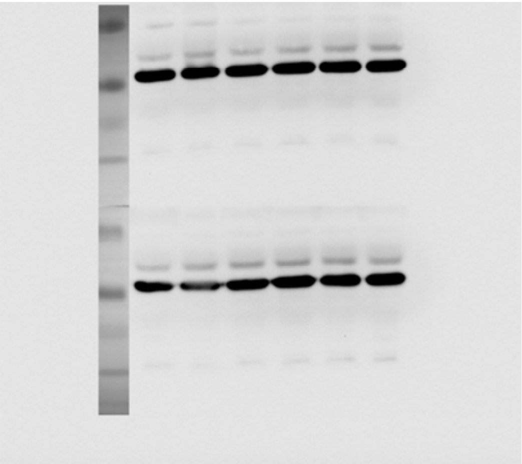

Fig. 3e p-c-Jun:

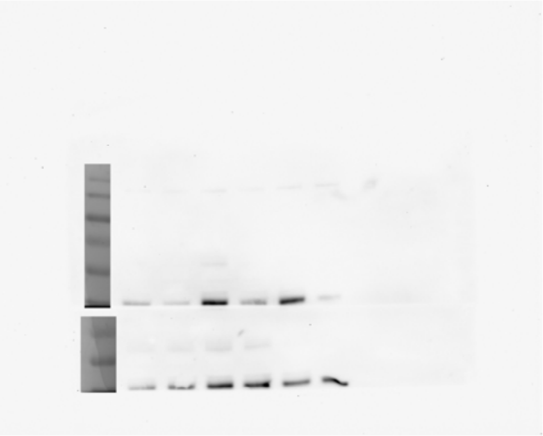

Fig. 3 e c-Jun:

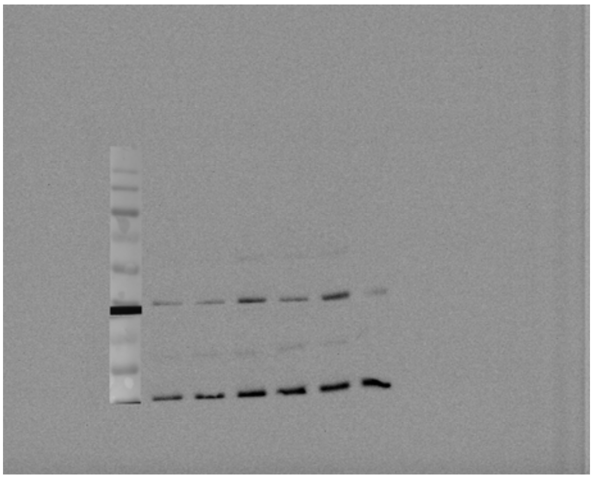

Fig. 3e Gapdh:

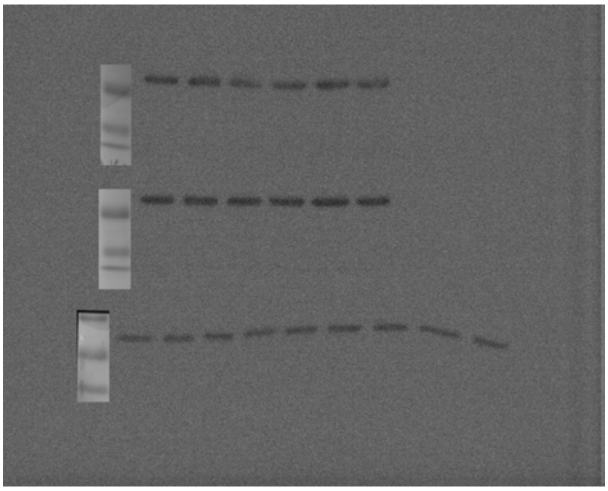

Fig. 4c Cyclin D1:

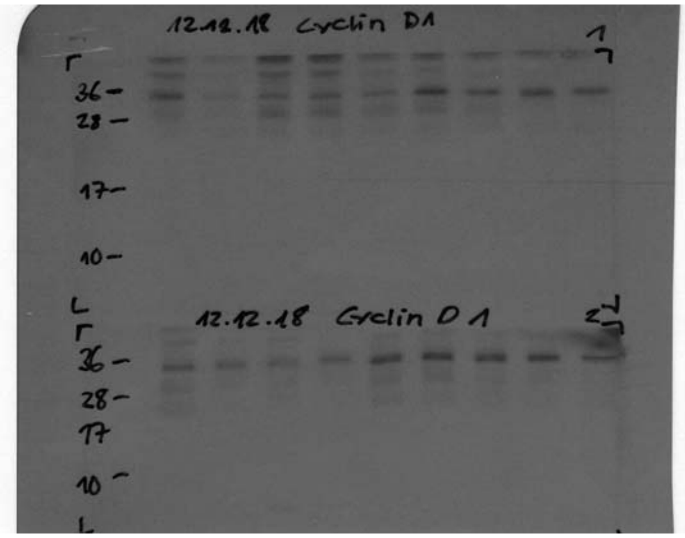

Fig. 4c Gapdh_a:

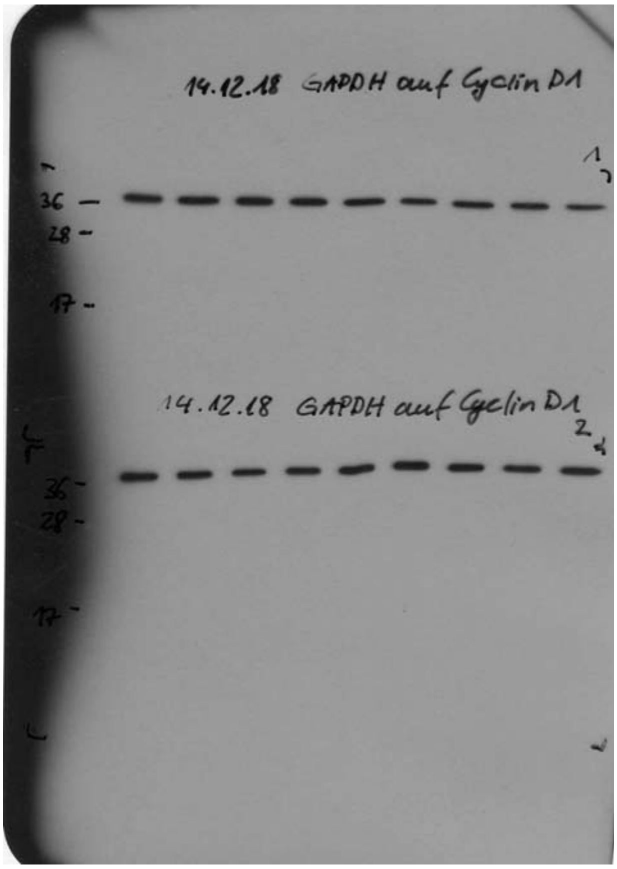

Fig. 4c Mcm2:

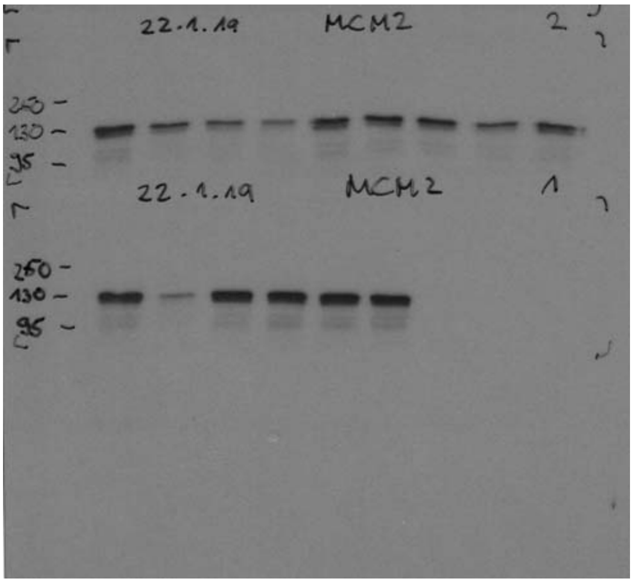

Fig. 4c Gapdh_b:

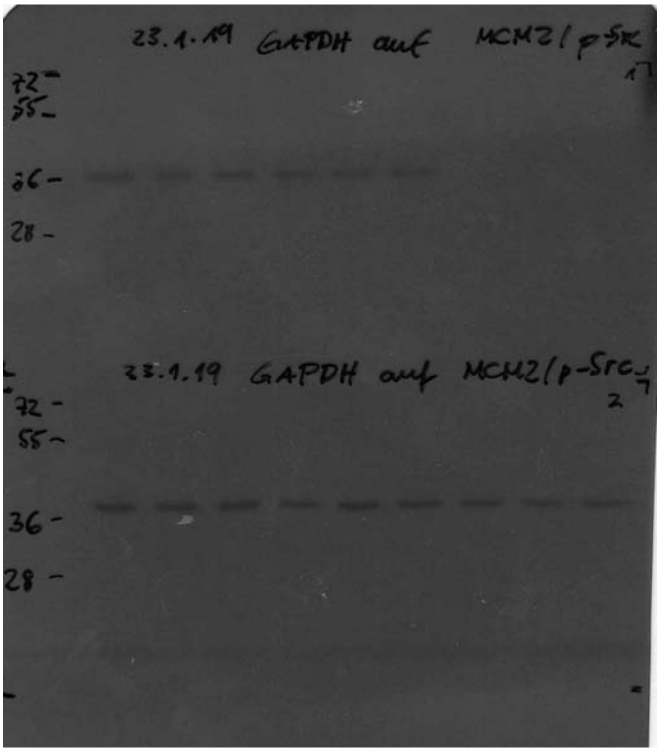

Fig. 4d Cyclin D1_a:

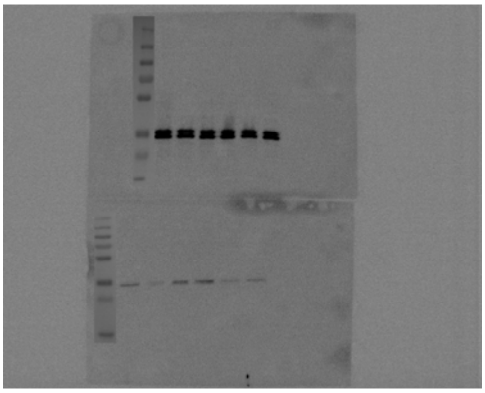

Fig. 4d α-Tubulin_a:

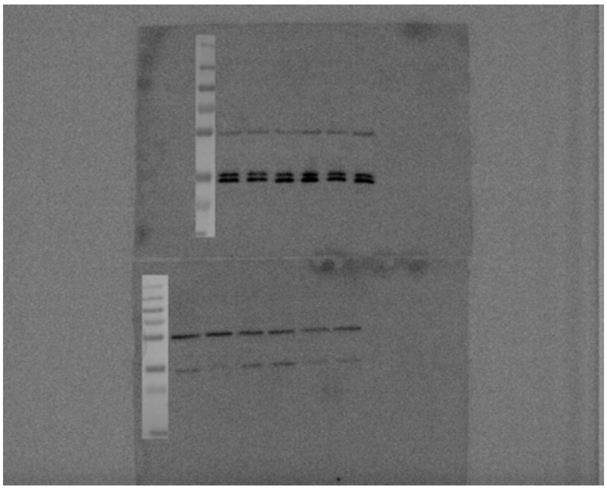

Fig. 4d Cyclin D1_b:

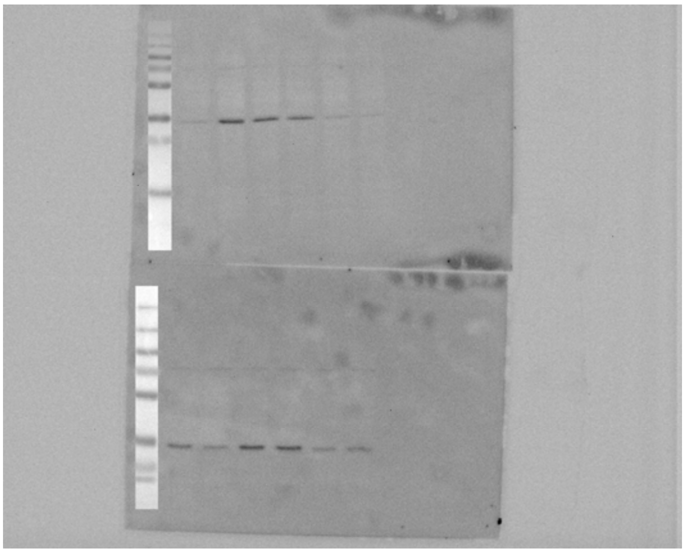

Fig. 4d α-Tubulin_b:

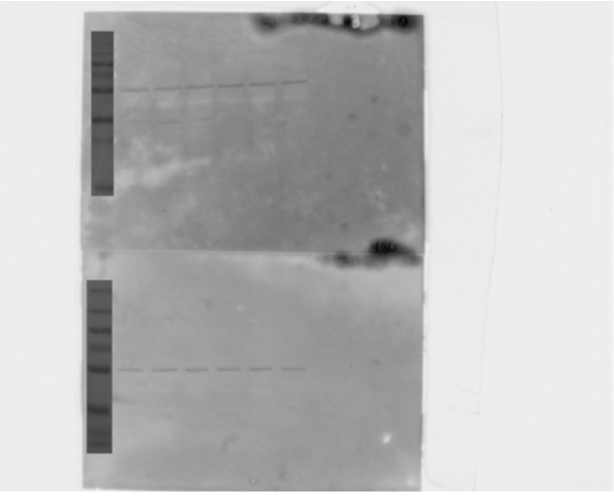

Fig. 4e Cyclin D1:

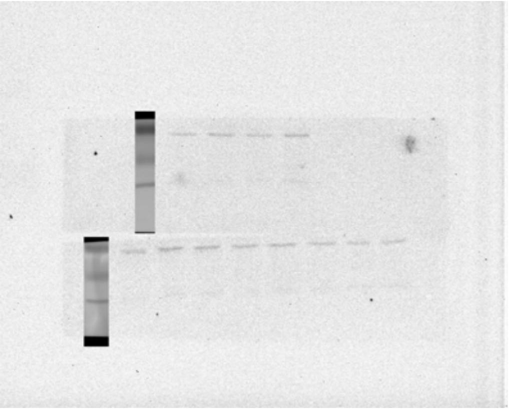

Fig. 4e α-Tubulin:

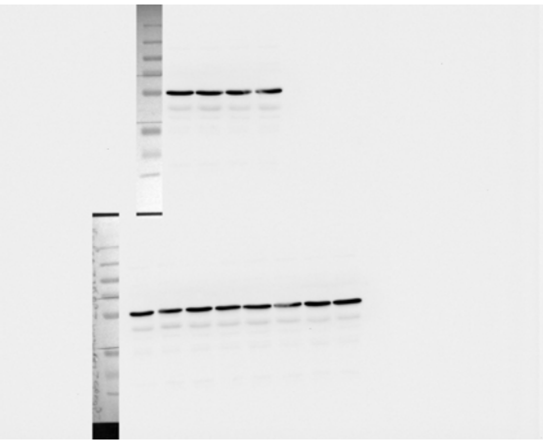

Fig. 4f Cyclin D1:

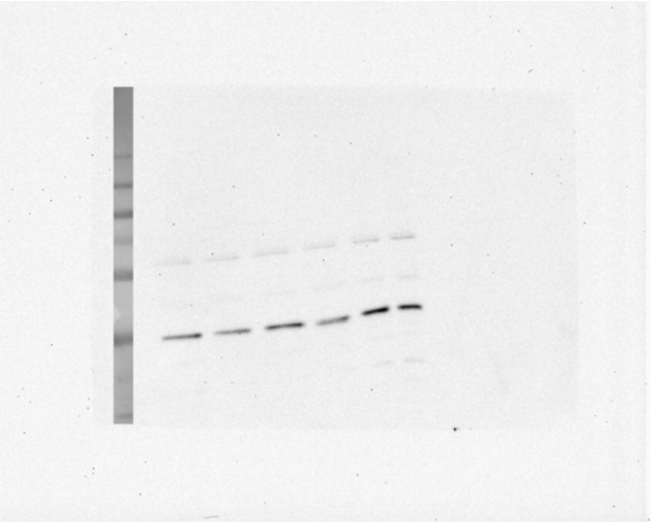

Fig. 4f Gapdh:

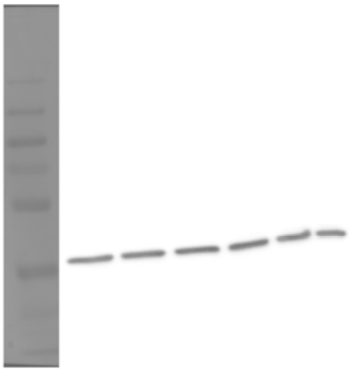

Fig. 5a γH2a.x:

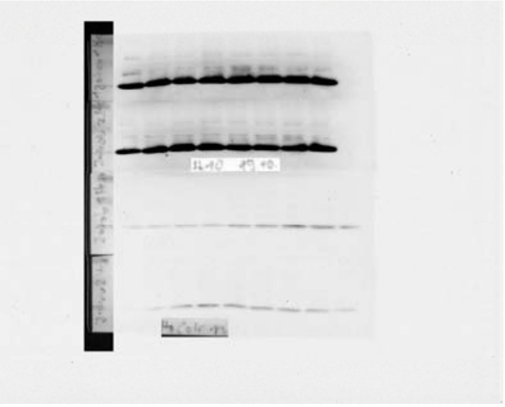

Fig. 5a Gapdh_a:

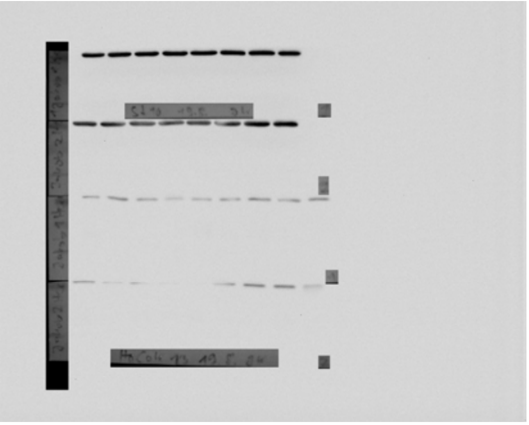

Fig. 5a Parp1:

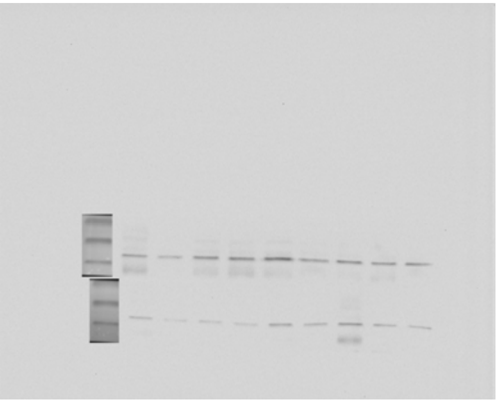

Fig. 5a Gapdh_b:

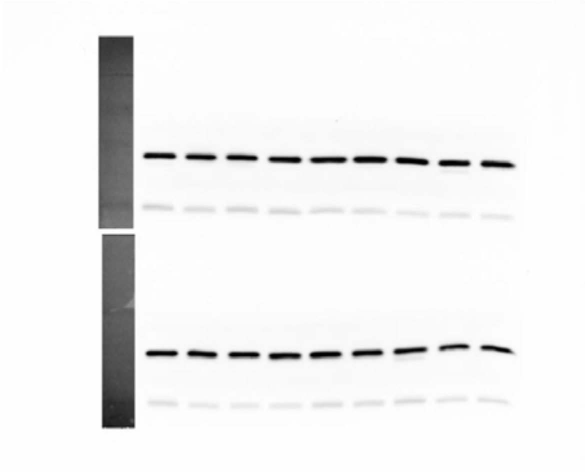

Fig. 5c γH2a.x:

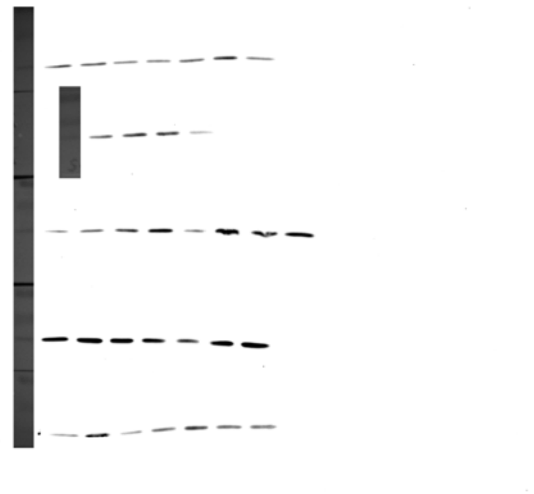

Fig. 5c Gapdh_a:

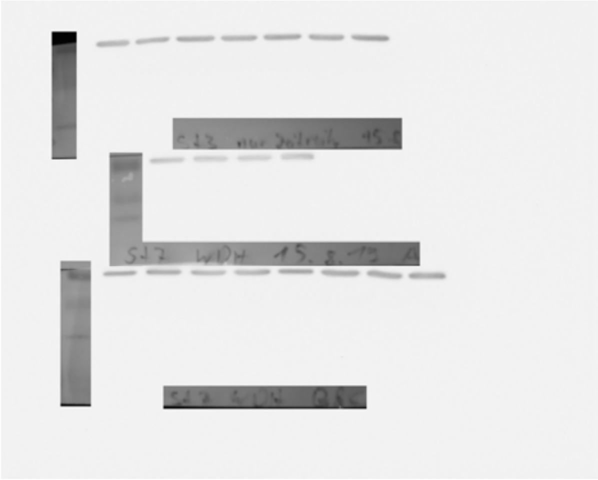

Fig. 5 c Parp1:

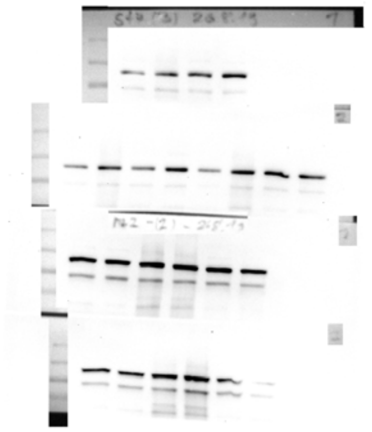

Fig. 5c Gapdh_b:

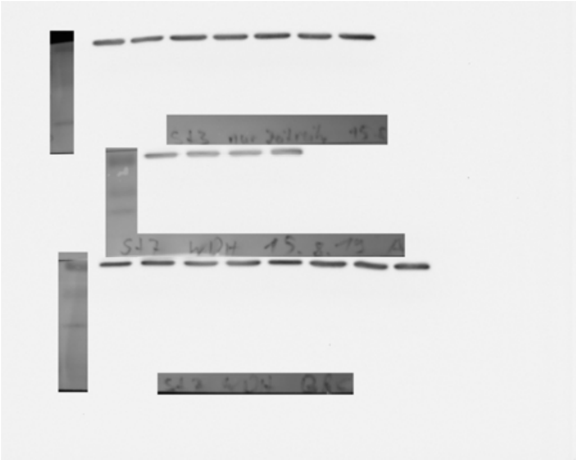

Suppl. Fig. 9 α-Tubulin:

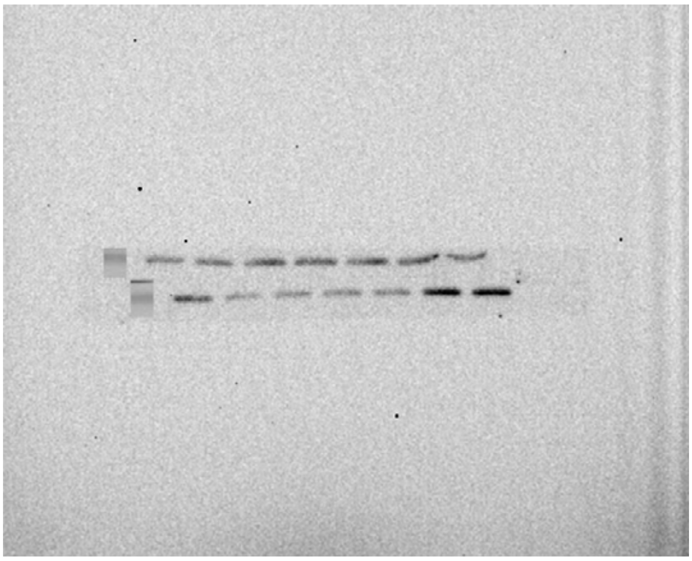

Suppl. Fig. 9 Cyclin D1:

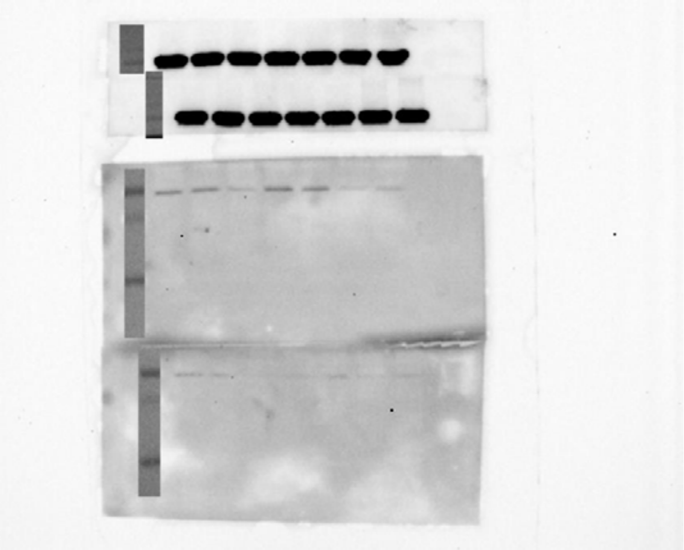

Supplement: Supplementary file 1 — Supplementary Information. [file 41598_2020_79450_MOESM1_ESM.pdf]
